# Brain-wide silencing of prion protein by AAV-mediated delivery of an engineered compact epigenetic editor

**DOI:** 10.1126/science.ado7082

**Published:** 2024-06-28

**Authors:** Edwin N. Neumann, Tessa M. Bertozzi, Elaine Wu, Fiona Serack, John W. Harvey, Pamela P. Brauer, Catherine P. Pirtle, Alissa Coffey, Michael Howard, Nikita Kamath, Kenney Lenz, Kenia Guzman, Michael H. Raymond, Ahmad S. Khalil, Benjamin E. Deverman, Eric Vallabh Minikel, Sonia M. Vallabh, Jonathan S. Weissman

**Affiliations:** 1 Whitehead Institute for Biomedical Research; Cambridge, MA 02142, USA.; 2 Department of Biological Engineering, Massachusetts Institute of Technology; Cambridge, MA 02139, USA.; 3 Howard Hughes Medical Institute, Massachusetts Institute of Technology; Cambridge, MA 02142, USA.; 4 Stanley Center for Psychiatric Research, Broad Institute of MIT and Harvard; Cambridge, MA02142, USA.; 5 Comparative Medicine, Broad Institute of MIT and Harvard; Cambridge, MA 02142, USA.; 6 Biological Design Center, Boston University; Boston, MA 02215, USA.; 7 Department of Biomedical Engineering, Boston University; Boston, MA 02215, USA.; 8 Wyss Institute for Biologically Inspired Engineering, Harvard University; Boston, MA 02115. USA.; 9 McCance Center for Brain Health and Department of Neurology, Massachusetts General Hospital; Boston, MA 02114, USA.; 10 Department of Biology, Massachusetts Institute of Technology; Cambridge, MA 02139, USA.; 11 David H. Koch Institute for Integrative Cancer Research, Massachusetts Institute of Technology; Cambridge, MA 02142, USA.

## Abstract

Prion disease is caused by misfolding of the prion protein (PrP) into pathogenic self-propagating conformations, leading to rapid-onset dementia and death. However, elimination of endogenous PrP halts prion disease progression. Here, we describe Coupled Histone tail for Autoinhibition Release of Methyltransferase (CHARM), a compact, enzyme-free epigenetic editor capable of silencing transcription through programmable DNA methylation. Using a histone H3 tail-Dnmt3l fusion, CHARM recruits and activates endogenous DNA methyltransferases, thereby reducing transgene size and cytotoxicity. When delivered to the mouse brain by systemic injection of adeno-associated virus (AAV), *Prnp*-targeted CHARM ablates PrP expression across the brain. Furthermore, we temporally limit editor expression by implementing a kinetically-tuned self-silencing approach. CHARM potentially represents a broadly applicable strategy to suppress pathogenic proteins, including those implicated in other neurodegenerative diseases.

## One-Sentence Summary

CHARM is an epigenetic editor that can be delivered by AAV to methylate DNA and silence prion protein throughout the brain.

## Introduction

Prion disease is caused by misfolding of the endogenous prion protein, PrP, initiating a chain reaction of templated misfolding to form toxic aggregates that cause neuronal death (1). PrP misfolding can occur spontaneously, the likelihood of which is increased by certain genetic mutations, or as the result of infection with misfolded prion seeds (1, 2). All subtypes of prion disease, including Creutzfeldt-Jakob disease (CJD), fatal familial insomnia (FFI), Kuru, and Gerstmann-Sträussler-Scheinker (GSS) disease in humans as well as scrapie, chronic wasting disease, and bovine spongiform encephalopathy (or mad cow disease) in animals, are caused by PrP (1). Despite the rarity of prion disease (3), our deep molecular understanding of its etiology provides a path toward potential treatment and prevention (2). Mice lacking *Prnp*, the gene encoding PrP, are resistant to prion infection (4) and depletion of PrP expressed in neurons after infection is sufficient to prevent prion disease progression and reverse symptoms in mice (5). Treatment of mice with antisense oligonucleotides (ASOs) targeting the *Prnp* transcript decreases expression of PrP and extends the survival of mice previously infected with misfolded PrP (6); however, the limited efficacy of ASOs and the requirement for chronic intrathecal dosing highlight the need for a more potent therapy. Importantly, both engineered and naturally occurring *PRNP* knockouts are well-tolerated in a variety of mammals (7–11). The only known knockout phenotype relates to disruption of a myelin maintenance signaling pathway (12) in which homozygous knockouts exhibit mild peripheral neuropathy (13, 14). These data indicate that strategies aimed at reducing PrP expression in neurons represent a viable therapeutic approach even after the onset of symptoms. Lessons learned in the development of this therapeutic approach may be applied to other neurodegenerative diseases, as there is now accumulating evidence that Parkinson’s, Alzheimer’s, Huntington’s, and other dementias involve protein aggregation as a central component of pathogenesis that can be targeted for therapeutic benefit (15, 16). Indeed, monoclonal antibodies targeting amyloid-beta plaques in early Alzheimer’s disease patients show a modest delay in cognitive decline (17).

Epigenetic silencing represents an attractive approach for eliminating expression of pathogenic proteins like PrP without the need to mutate the underlying DNA sequence (18–24). Permanent silencing can be achieved through targeted DNA methylation by the recruitment of the constitutively active catalytic domain (D3A) of the de novo DNA methyltransferase enzyme DNMT3A (25–27) along with the C-terminal domain (D3L) of its cofactor DNMT3L (28–30). DNA methylation at cytosine-guanine dinucleotide (CpG) sites, producing 5-methyl-CpGs (5mCpGs), is mitotically inherited and contributes to transcriptional silencing directly by blocking transcription factor binding and indirectly by recruiting methyl-CpG-binding factors that induce heterochromatin (31). These domains, with the addition of a repressive KRAB domain, were fused to a nuclease-deficient *S. pyogenes* Cas9 (dSpCas9) yielding a CRISPR-based editor for programmable, heritable gene silencing termed CRISPRoff (32). CRISPRoff has the benefit of a wide effective targeting window at gene promoters due to CpG methylation spreading, and its effect is generally stable through cell division and differentiation. This stands in contrast to CRISPRi (dCas9-KRAB), which enables robust but transient gene repression (18). Additionally, genome-wide screening indicated that the large majority of genes can be silenced with CRISPRoff (32). Prion disease is an excellent candidate for this approach, since simply decreasing PrP expression will have a therapeutic effect (2) and the human *PRNP* promoter contains a large annotated CpG island to serve as a substrate for DNA methylation. However, the complexity of the CRISPRoff system leads to challenges for delivery and toxicity as a therapeutic and necessitates the development of a more compact, potent, and safe epigenetic silencer.

## Results

### The prion gene is a viable target for durable epigenetic silencing.

**Fig. 1.**
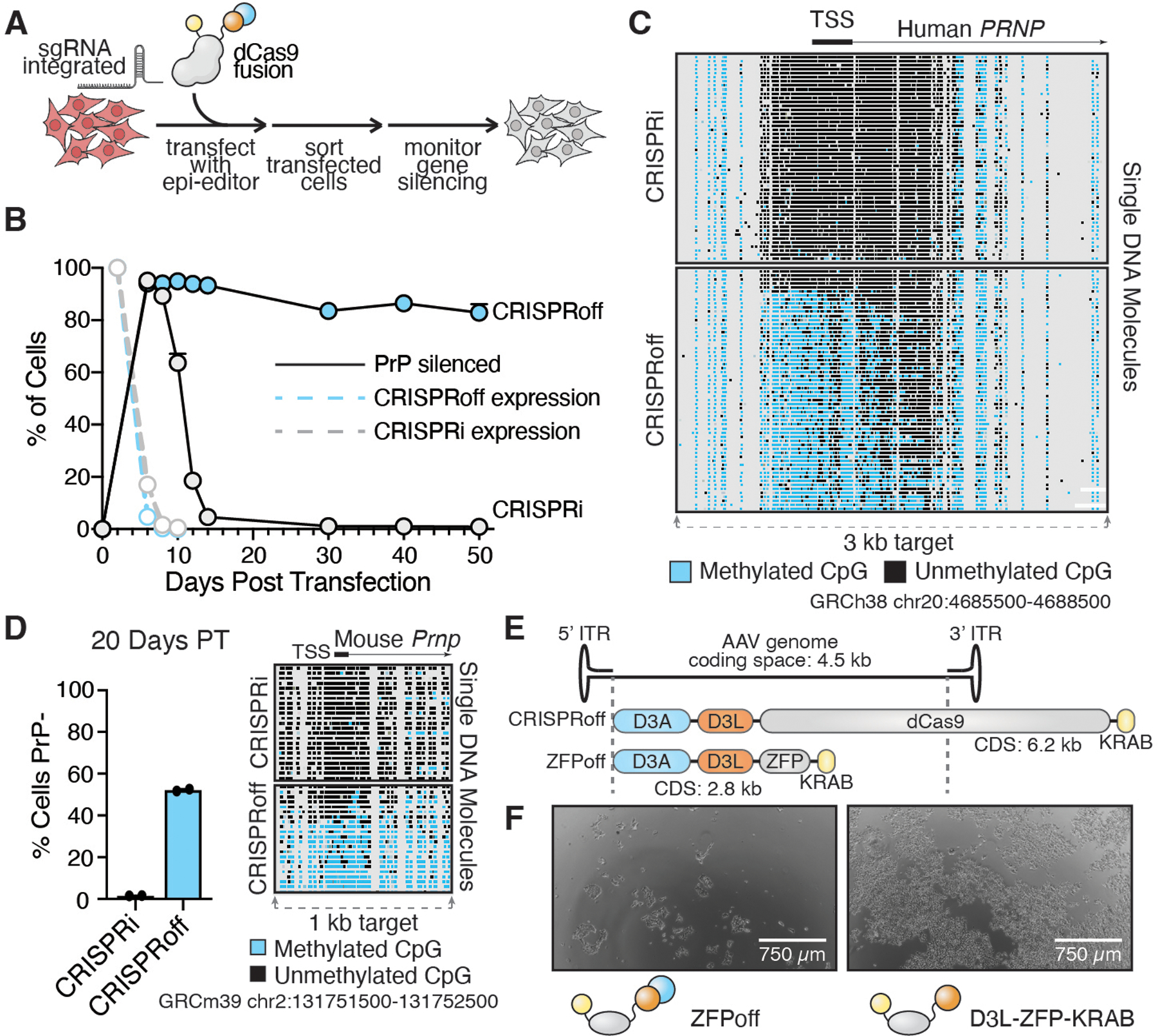
*PRNP* is a viable target for epigenetic silencing, but existing technologies are not suitable for therapeutic use. (**A**) A HEK293T cell line was made by integrating a lentiviral vector containing mU6-sgRNA targeting the *PRNP* TSS. Transfected cells were sorted by FACS (TagBFP) two days post-transfection and monitored for *PRNP* silencing by Alexa Fluor 647 anti-PrP staining and flow cytometry. (**B**) PrP and effector expression time course of HEK293T cells transiently transfected with plasmids encoding CRISPRi and CRISPRoff effectors. Data are mean ± SEM of n=2 replicates. (**C**) DNA methylation assessment by targeted nanopore long read sequencing of native genomic DNA extracted from HEK293T cells 50 days post-transfection. (**D**) Mouse N2a cells co-transfected with plasmids encoding CRISPRi/CRISPRoff and three sgRNAs targeting the TSS of *Prnp* were assessed for PrP expression and DNA methylation. (**E**) Schematic depicting AAV genome packaging constraints with CRISPRoff and ZFPoff to scale. (**F**) HEK293T cells were transiently transfected with ZFPoff and D3L-ZFP-KRAB and imaged after 6 days.

We first assessed the suitability of the mouse and human *PRNP* gene to epigenetic silencing with targeted DNA methylation using CRISPRoff. We transduced HEK293T cells with a single guide RNA (sgRNA) targeting the transcription start site (TSS) of the *PRNP* gene. CRISPRoff and CRISPRi (18) were introduced by transient transfection and PrP expression was assessed by flow cytometry with fluorescent anti-PrP antibodies (Fig. 1A). With a transient pulse of CRISPRoff effector, *PRNP* remains durably silenced for at least 50 days. As expected, the repressive effect of CRISPRi was reversed rapidly upon loss of effector expression (Fig. 1B). Target-enriched nanopore sequencing of native DNA confirmed extensive multi-kilobase (kb) DNA methylation across the CpG island of the *PRNP* promoter region with the CRISPRoff treatment but not with CRISPRi (Fig. 1C). Similarly, targeting of CRISPRoff to mouse *Prnp* in Neuro-2a (N2a) cells led to silencing and DNA methylation (Fig. 1D, fig. S1), confirming that mice would be a viable model for in vivo prion repression experiments using our epigenetic editor (epi-editor; see below).

### Existing epigenome editors are too large or too toxic for therapeutic use.

In its current form, CRISPRoff is poorly suited to be a therapeutic for prion disease. The preferred vehicle for transgene delivery to the central nervous system (CNS) is the adeno-associated virus (AAV), which can be efficiently packaged with cargo around 4.7 kb in length including inverted terminal repeats (33). The D3A-D3L-dCas9-KRAB fusion comprising CRISPRoff is ~6.2 kb long, far exceeding the packaging capacity of an AAV vector (Fig. 1E). Most of this space is occupied by the 4.1 kb coding sequence of dSpCas9. Moreover, because AAV genomes form concatenated episomes that chronically express the transgene, the bacterial enzyme Cas9 is likely to become antigenic over time (34, 35)— a large proportion of the human population already has an immune memory of it (36). To overcome these obstacles, dCas9 can be replaced with a different DNA-binding modality.

Zinc finger proteins (ZFPs) are ubiquitous DNA-binding proteins in eukaryotes (37) whose modular nature has enabled programming for specific genome targeting (38–42). ZFPs offer some advantages as a therapeutically-relevant DNA targeting module; their compact size, roughly an order of magnitude smaller than that of SpCas9, makes them suitable for delivery via AAV (Fig. 1E), and they are also less immunogenic due to their lack of bacterial epitopes (43). Previous work has motivated our interest in using ZFPs in our effector constructs for targeted and heritable gene silencing, since engineered ZFPs fused to chromatin-modifying domains have successfully modulated gene transcription in vivo as long as they are continuously expressed (44–47).

The next challenge to overcome is cytotoxicity. The full length de novo DNMT3 methyltransferases are regulated by an autoinhibitory mechanism (48), which CRISPRoff bypasses by only using the catalytically-active methyltransferase domain D3A (32). The D3A domain on its own can have detrimental effects when overexpressed in target cells; indeed, a ZFPoff construct transiently overexpressed in HEK293 cells exhibited substantial cytotoxicity whereas cells transfected by the same ZFP fusion without the D3A catalytic domain recovered quickly (Fig. 1F and see below). Attempts to instead recruit full length DNMT3A for DNA methylation have previously been described (49). Accordingly, we tried using a single GFP nanobody (50) to bind EGFP-tagged DNMT3A, a strategy which has been efficacious for the recruitment of other repressive domains (51), but we observed poor activity (fig. S2A and B).

The dominant de novo methyltransferase in somatic tissues, particularly in the brain, is the isoform DNMT3A1, whereas DNMT3B is virtually nonexistent (52); all mentions of DNMT3A hereby refer to DNMT3A1. DNMT3A normally exists in an autoinhibited conformation in which its methyltransferase domain is occluded by its ADD domain, and this is only released upon binding of the ADD domain to unmethylated histone H3 lysine 4 (H3K4me0), which is methylated near active promoters resulting in DNAme inversely correlated with H3K4 methylation (53–55). This mechanism couples DNMT3A activity to its two chromatin-reading domains: the ADD domain which reads H3K4me0 and the PWWP domain which reads di- and trimethylated histone H3 lysine 36 (H3K36me2/3) enriched in transcribed gene bodies (48) (fig. S3A). In vitro, purified DNMT3A is stimulated by a 12 amino acid H3K4me0 peptide to methylate a substrate (53, 55, 56). Furthermore, DNMT3A complexes with DNMT3L in a 2:2 stoichiometry through hydrophobic contacts in their C-terminal domains (57). This suggests that DNMT3L, which is catalytically inactive and has an ADD domain of its own (fig. S3B), may help stabilize DNMT3A and could assist the active methyltransferase in seeking an appropriate target for DNAme (58). DNMT3L co-immunoprecipitation experiments indicate that it interacts with both DNMT3A and DNMT3B to coordinate de novo DNAme (59). These interactions are the key to exploiting the endogenous pool of de novo methyltransferases for epigenetic editing strategies, thus removing the need to overexpress toxic quantities of the D3A catalytic domain.

### A fusion of the histone H3 tail and Dnmt3l C-terminal domain efficiently mediates heritable gene silencing in cells.

**Fig. 2.**
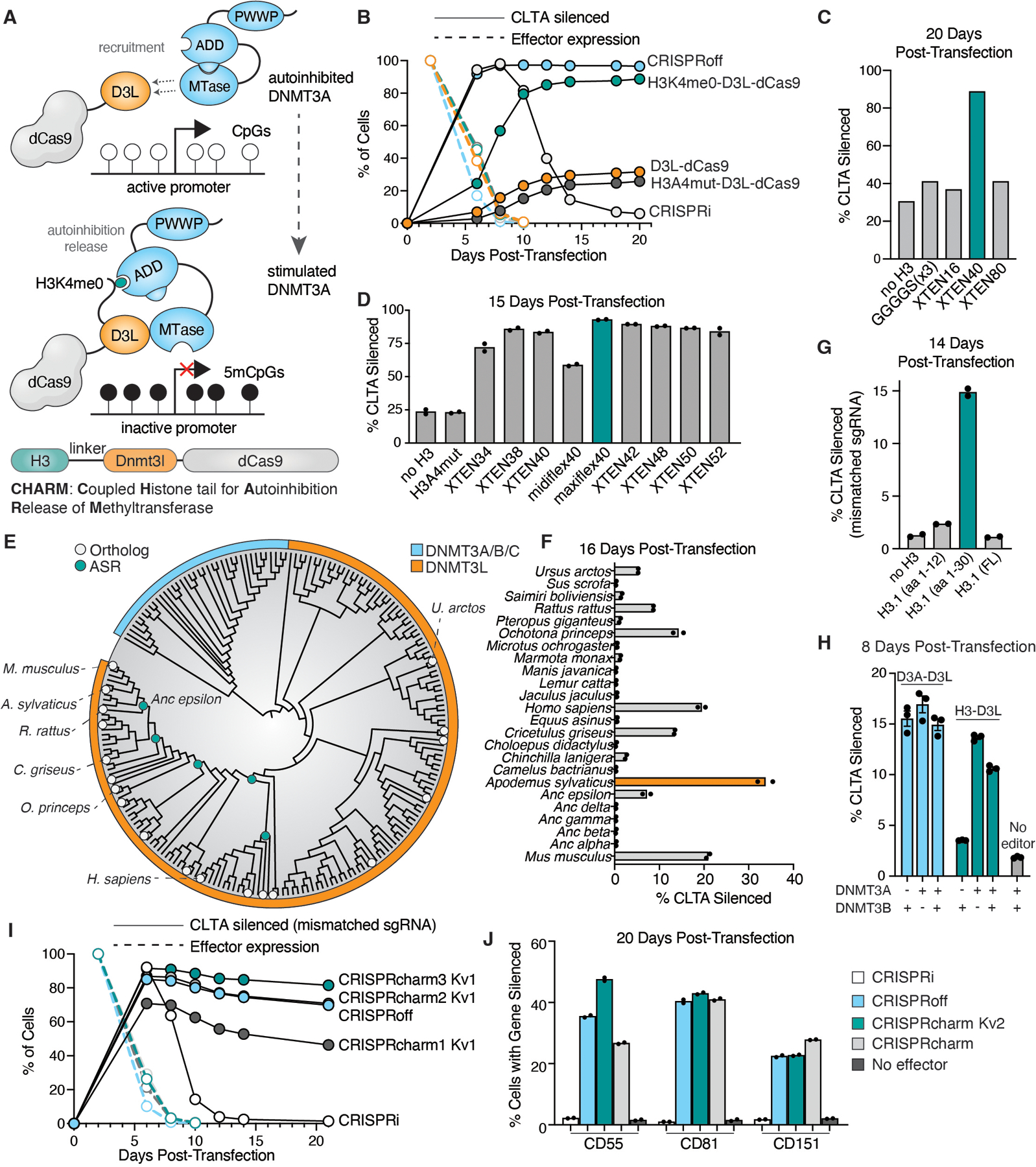
A histone H3 tail fused to the Dnmt3l C-terminal domain acts as a potent mediator of DNA methylation and transcriptional silencing. (**A**) Cartoon depiction of endogenous DNMT3A recruitment and activation by the CHARM system. (**B**) Time course of effector (TagBFP) and mScarlet-*CLTA* reporter expression after transient transfection with effector-containing plasmids. Data are mean ± SEM of n=2 replicates. (**C**) First pass histone H3 tail fusion test on the mScarlet-*CLTA* reporter using different linkers to D3L. (**D**) Refinement of linker sequence between H3 tail and D3L. (**E**) Phylogenetic tree of DNMT3L orthologs and ancestral reconstruction nodes. Orthologs with measured silencing activity >5% by 14 days are labeled. **(F**) Repression of mScarlet-*CLTA* reporter 2 weeks post-transfection with different DNMT3L ortholog C-terminal domains fused to dCas9. (**G**) Transient transfection and repression of mScarlet-*CLTA* reporter with different length histone isoform H3.1 domains (FL; full length). A mismatched sgRNA against *CLTA* TSS is used to improve dynamic range. (**H**) Transient transfection of sgRNA and effector fused to dCas9 targeting the *CLTA* reporter in a methyltransferase knockout background. Data are mean ± SEM of n=3 replicates. (**I**) Time course of effector expression and mScarlet-*CLTA* silencing comparing CRISPRoff and CRISPRi against the series of optimized CHARM constructs. Data are mean ± SEM of n=2 replicates. (**J**) Comparison of CRISPRi, CRISPRoff, and the optimized CRISPRcharm effectors in silencing cell surface markers. Vectors encoding mU6-sgRNAs were transduced via lentivirus and effector plasmids were transiently transfected.

Taking advantage of the known interactions between DNMT3A, DNMT3L, and H3K4me0, we developed a new strategy for targeted DNA methylation and epigenetic silencing, analogous to but distinct from CRISPRoff, by leveraging the use of the endogenous methyltransferases in cells. Rather than overexpressing the D3A methyltransferase domain as a fusion protein, we instead recruit the full length enzyme to the target site through interactions with the D3L domain and stimulate its activity using an unmethylated H3 tail fused to the N-terminus of the epi-editor. We named this new effector CHARM: Coupled Histone tail for Autoinhibition Release of Methyltransferase (Fig. 2A).

Using the *CLTA* gene tagged with mScarlet as a fluorescent reporter for endogenous gene silencing in HEK293T cells, we systematically compared several epi-editors including the canonical CRISPRoff and CRISPRi constructs. As expected, CRISPRoff silenced the reporter durably, CRISPRi repressed the reporter transiently, and D3L-dCas9 had a minimal silencing effect. The 12 aa H3K4me0 peptide fused to D3L-dCas9 resulted in silencing almost on par with CRISPRoff despite lacking the KRAB domain. To demonstrate that the unmethylated H3K4 residue is critical for endogenous DNMT3A stimulation, we mutated the lysine to alanine (H3A4). This mutant resulted in no silencing improvement over D3L-dCas9 alone (Fig. 2B). The H3 tail fusion was particularly sensitive to linker modifications connecting it to the D3L domain; in the first round of testing, only a 40 amino acid linker could achieve robust silencing (Fig. 2C). We further show the importance of the autoinhibition release mechanism of the CHARM system by testing full length DNMT3A direct fusions on our *CLTA* reporter where only the truncated D3A catalytic domain achieved the full silencing effect analogous to histone tail mediated activation (fig. S3C).

To verify that this effect is due to the mechanism of histone tail binding the ADD domain of the methyltransferase rather than simply stabilizing or altering expression of the fusion protein, we performed a transfection dose titration comparing D3L with or without the H3 tail. At 18 days post-transfection, there was little difference in silencing activity across transfected DNA concentrations, indicating that the epi-editor is unlikely to be dose-limited (fig S4A). Likewise, gating for different levels of expression while sorting the transfected cells did not improve silencing efficacy by day 16 post-transfection (fig. S4B). This indicates that activity of the epi-editor is not limited by translation or stability of the fusion protein.

### CHARM optimization

We next sought to optimize the CHARM effector by manipulating various parameters of the fusion protein. First, we tested a range of linker lengths centered around the established 40 amino acid length. We also modified the canonical XTEN linker amino acid sequence to increase the flexibility through the removal of proline residues to generate the “midiflex” and “maxiflex” linker variants following general linker engineering guidelines (60). The 40 amino acid maxiflex linker provided a modest increase in silencing activity, and this epi-editor was denoted CRISPRcharm1 (Fig. 2D). Attempts to boost activity by increasing nuclear localization through the addition of an N-terminal nuclear localization signal (NLS) or by appending two H3 tails in tandem were unsuccessful (fig. S4C), suggesting that having a free N-terminus is critical for CHARM function. Furthermore, fusions of either the H3 tail or D3L alone to dCas9 had lower silencing capability than the two together, indicating a synergistic rather than additive effect (fig. S4D).

Another variable to optimize was the D3L domain sequence, which is critical for methyltransferase recruitment and stabilization. Rather than performing random mutagenesis, we restricted our exploration to the extant universe of D3L domains orthologous to the canonical *Mus musculus* D3L, as well as some ancestral reconstructions (ASRs) (61) between the rodent and primate clades (Fig. 2E). Approximately two dozen D3L orthologs and ASRs fused to dCas9 were tested on the *CLTA* reporter, with the most active being the D3L domain of the European wood mouse *Apodemus sylvaticus* (AsD3L) (Fig. 2F). This epi-editor became CRISPRcharm2.

While the first 12 amino acids of the histone H3 tail were sufficient to stimulate methylation activity, we speculated that a longer portion of the flexible tail region of histone H3.1 could have a higher affinity for the ADD domain of DNMT3A. When recruited to our *CLTA* reporter using a mismatched sgRNA to avoid saturation of the transcriptional silencing signal, we found that a 30 amino acid H3 tail, but not the full H3.1 protein including the globular domain, resulted in a more potent CHARM effector (Fig. 2G); this was designated CRISPRcharm3. Consistent with the proposed mechanism of CHARM, we found that CHARM requires DNMT3A (and not DNMT3B) by knocking out the de novo methyltransferases in our *CLTA* reporter cells, whereas this genetic background did not impact silencing efficacy with exogenously supplied D3A (Fig. 2H and fig. S4E). These optimization efforts led to a CHARM effector exceeding the transcriptional silencing capabilities of CRISPRoff (Fig. 2I) when a KRAB domain was fused to the C-terminus of dCas9, as in CRISPRoff (32). Schematics of each of these CHARM epi-editors can be found in figure S4F, with the optimized version without the KRAB domain hereby referred to as CRISPRcharm.

We further observed that the KRAB domain could be incorporated into the flexible linker between the H3 tail and D3L. The two KRAB domain-containing variants were named CRISPRcharm Kv1 and Kv2, respectively, and both can efficiently repress the *CLTA* reporter (fig. S4G). To test the breadth of targeting capabilities of our new CHARM effector, we targeted CRISPRoff, CRISPRcharm, and CRISPRcharm Kv2 to three cell surface markers using HEK293T cells with pre-integrated sgRNAs. Antibody staining and flow cytometry revealed durable repression out to about three weeks on par with CRISPRoff (Fig. 2J), suggesting that CHARMs will be effective on the broad range of genes amenable to DNA methylation-mediated silencing (32).

### CHARMs are compatible with different DNA-binding domains.

**Fig. 3.**
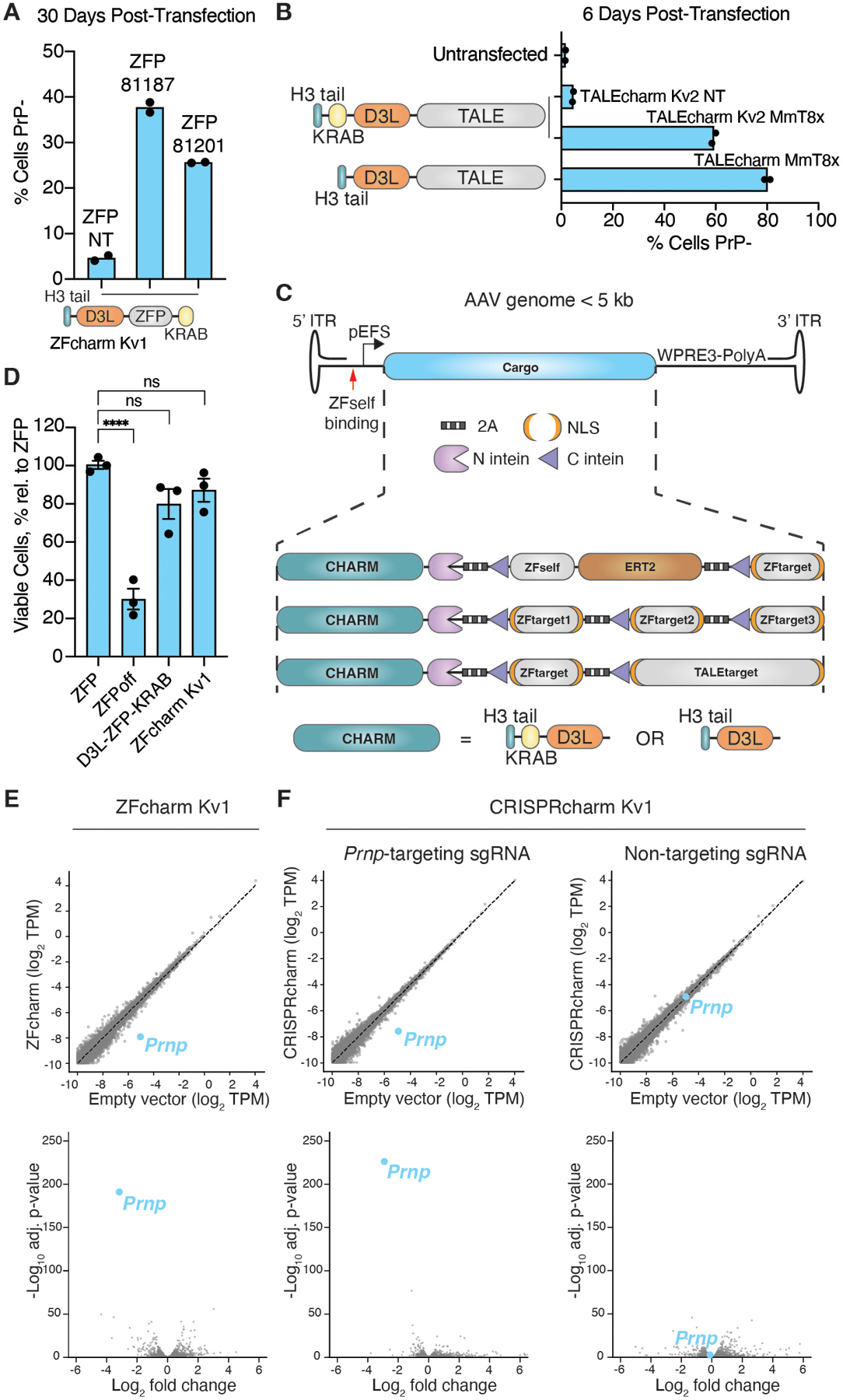
CHARM is flexible and specific. (**A**) Mouse N2a cells were transiently transfected with plasmids encoding ZFcharm Kv1 constructed with the mouse *Prnp*-targeting ZFP 81187, ZFP 81201, or a non-targeting ZFP and stained with Alexa Fluor 647 anti-PrP. (**B**) Mouse N2a cells were transiently transfected with TALEcharm and TALEcharm Kv2 composed of engineered TALE proteins targeting the mouse *Prnp* TSS or a non-targeting TALE, then measured using Alexa Fluor 647 anti-PrP. (**C**) Schematic of AAV packaging using space-saving techniques like split-inteins (65) or a self-silencing approach including the tamoxifen-inducible engineered estrogen receptor ERT2 (66). WPRE3 is a structured 3’ element for mRNA stability (67). (**D**) HEK293T cells were transiently transfected with plasmids encoding ZFP81187 alone or the fusions ZFPoff, D3L-ZFP-KRAB, and ZFcharm Kv1 and then counted by flow cytometry after cell viability staining with LIVE/DEAD near-IR dye. Data are mean ± SEM of n=3 replicates. Statistical analyses are one-way ANOVAs followed by Tukey’s multiple comparisons test (**** p<0.0001; ns, not significant). **(E** and **F)** ZFcharm Kv1 (**E**) and CRISPRcharm Kv1 (**F**) were introduced into N2a cells by lentiviral transduction and assessed for *Prnp* knockdown and specificity by RNA sequencing 4 weeks later. CRISPRcharm Kv1 was evaluated in N2a cells expressing sgRNA targeting the mouse *Prnp* TSS or a non-targeting sgRNA. Log_2_ fold changes (upper) and volcano plots (lower) were generated from DESeq2.

After optimizing the CHARM effector using CRISPR-dCas9 recruitment to our endogenous *CLTA* reporter, we next explored the potential of using different DNA-binding modalities to reduce transgene size and facilitate packaging into an AAV vector. We replaced dCas9 in CRISPRcharm Kv1 with previously published ZFPs developed by Sangamo Therapeutics targeting the mouse *Prnp* promoter (ZFPs 81187 and 81201) (62) to generate ZFcharm Kv1. These were transiently transfected into N2a cells and achieved durable (> 1 month) PrP silencing (Fig. 3A). Transcription activator-like effectors (TALEs), another mode of programmable DNA-binding domain, have also been shown to enable targeted DNA methylation (29). TALEs can be easier to design than ZFPs, so we constructed chimeric TALEs (63) fused to the CHARM effector (denoted TALEcharm) to target the mouse *Prnp* promoter, with some success (Fig 3B).

The relatively small size of ZFcharms and TALEcharms enables flexible single-vector AAV packaging strategies with the potential for multiplexed targeting. Inspired by recent work for AAV delivery of base editors (64), we leveraged the split *Nostoc punctiforme* (Npu) intein strategy for trans-splicing (65) of polypeptides. We constructed ZFcharms with the CHARM effector (with N-terminal Npu intein) separated from the ZFP DNA-binding domain (with C-terminal Npu intein) by 2A ribosome skipping sequences, thus generating two distinct polypeptide chains from the same mRNA transcript. These polypeptides are spliced together to form the complete ZFcharm Kv2 molecule and are as effective at silencing *PRNP* as direct fusions (fig. S5A). Building on this idea, we propose multiple AAV cargo designs that fall within the 5 kb AAV genome limit and enable multiplexed targeting by distinct DNA-binding domains while only encoding a single CHARM effector in the transgene. The compact size even allows more sophisticated approaches for small molecule control of gene silencing, such as through inducible nuclear localization of an engineered estrogen receptor when bound to tamoxifen (66) (Fig. 3C).

For the remaining studies, we focus on the use of ZFPs for DNA target engagement. However, we observed efficient targeting and gene silencing with both *S. pyogenes* CRISPR-Cas9 and TAL effector modalities. It is thus highly likely that CHARM effectors will be broadly compatible with other DNA-binding domains; indeed, we demonstrate efficient silencing of our *CLTA* reporter using a nuclease-deficient version of the smaller *S. aureus* Cas9 (dSaCas9) more amenable to AAV packaging (68) (fig. S5B).

### CHARMs exhibit low toxicity with high specificity.

We observed marked cytotoxicity associated with transient overexpression of ZFoff, but not with D3L-ZFP-KRAB lacking the catalytic D3A domain (see above). This led us to hypothesize that ZFcharms, which replace D3A with a short histone tail peptide and have no catalytic activity on their own, would be better-tolerated in cells. By transiently transfecting HEK293T cells, performing FACS to isolate transfected cells, and then staining and counting cells with a viability dye, we quantified the cytotoxicity of ZFcharm Kv1 expression. ZFoff-transfected cells were significantly less viable six days after transfection, whereas ZFcharm Kv1-transfected cells were indistinguishable from cells transfected with ZFP lacking any effector domains (Fig. 3D).

To assess the specificity of ZFcharm Kv1, we performed RNA sequencing 28 days post-transduction of N2a cells by lentivirus containing ZFcharm Kv1 targeting *Prnp* and saw minimal off-target gene repression (Fig. 3E). This is consistent with our analysis of N2a cells transduced with CRISPRcharm Kv1 and sgRNA targeting *Prnp* or with a non-targeting sgRNA (Fig. 3F), suggesting that CHARM expression has minimal bystander effects and its specificity is largely dictated by the DNA-binding domain. Likewise, we quantified the knockdown of *Prnp* transcripts and saw nearly complete repression when compared to non-targeting or effector-null conditions (fig. S6).

### AAV-delivered CHARMs lead to brain-wide Prnp repression and methylation.

**Fig. 4.**
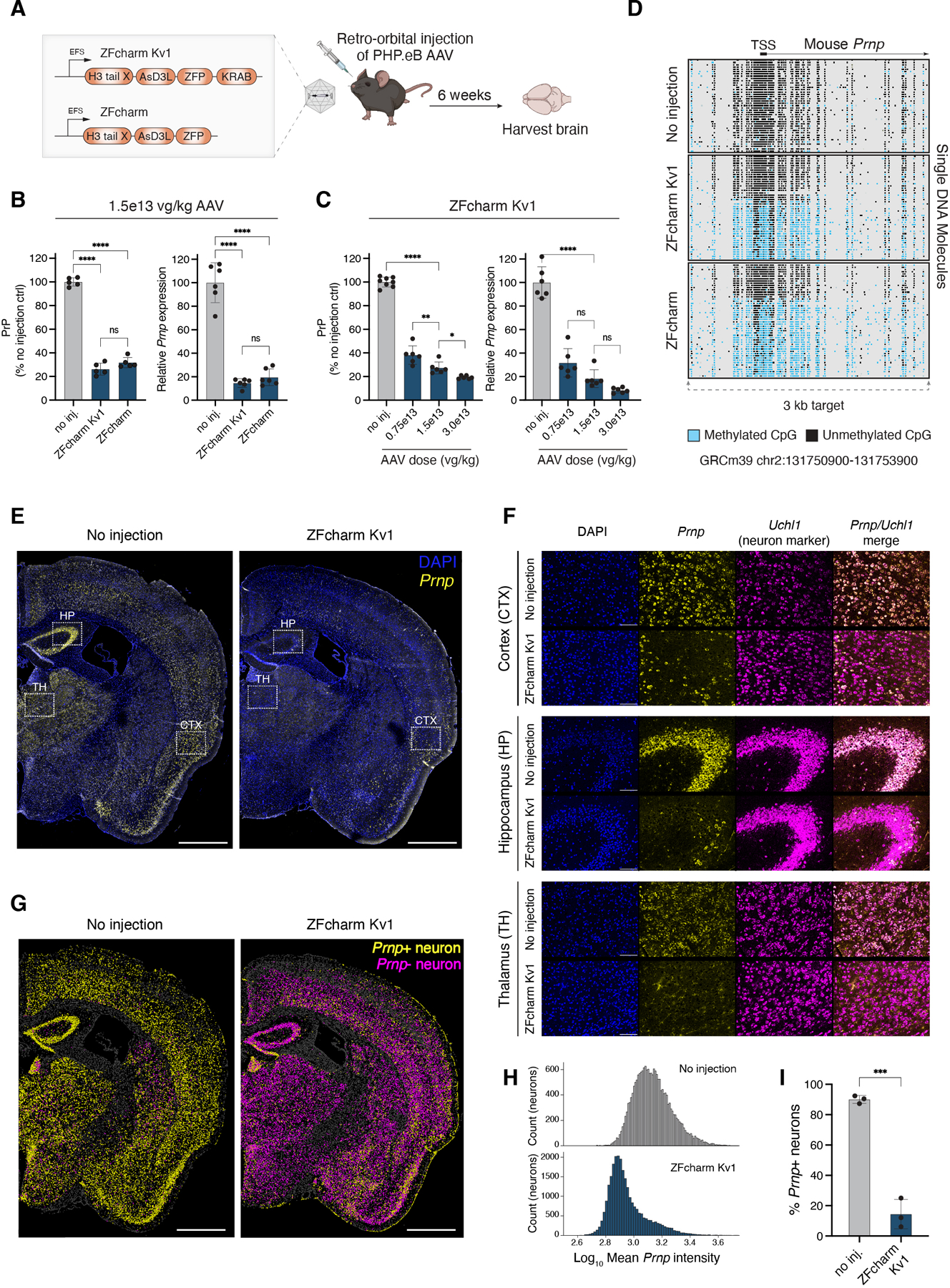
AAV-delivered ZFcharms repress and methylate *Prnp* in vivo. **(A)** Schematic of experimental design. ZFcharm Kv1 and ZFcharm were delivered to mice via AAV and whole brains were harvested 6 weeks later. Unless otherwise noted, the AAV dose was 1.5e13 vg/kg. **(B)** PrP ELISA and *Prnp* RT-qPCR data generated from brain hemisphere homogenate 6 weeks post injection. Data are mean ± SD of n=5–6 replicates. **(C)** ZFcharm Kv1 AAV dose-response analysis. Data are mean ± SD of n=6–8 replicates. **(D)** Quantification of DNA methylation via nanopore sequencing of the *Prnp* promoter. **(E** and **F)** Visualization of *Prnp* (yellow) and pan-neuronal marker *Uchl1* (magenta) expression in coronal brain sections via HCR RNA-FISH (DAPI staining in blue). **(E)** Representative maximum-intensity projections of coronal brain hemisphere tile scans. White boxes indicate brain regions shown in panel F. Scale bar, 1mm. **(F)** Zoomed-in views of the cortex (CTX), hippocampus (HP), and thalamus (TH). Scale bar, 100 µm. **(G)** Machine learning classification of *Prnp*+ (yellow) and *Prnp*- (magenta) neurons using QuPath software (71). *Uchl1*- cells are shown in gray. Cell boundaries represent 4 µm expansions from DAPI-detected nuclei. Scale bar, 1mm. **(H)** Representative histograms of mean *Prnp* intensity in neurons. **(I)** Bar chart showing % *Prnp*+ neurons in treated and untreated brains based on QuPath classification. Data are mean ± SD of n=3 replicates. Statistical analyses are one-way ANOVAs followed by Tukey’s multiple comparisons test for panels B and C, and unpaired t test for panel I (* p < 0.05; ** p < 0.005; *** p < 0.0005; **** p<0.0001; ns, not significant).

Having achieved CHARM-mediated heritable *Prnp* silencing in cultured cells, we tested silencing efficacy in vivo through AAV delivery to the mouse brain. Constructs with and without the KRAB domain (ZFcharm Kv1 and ZFcharm, respectively) were packaged into AAV-PHP.eB, a capsid engineered for high transduction efficiency throughout the mouse CNS (69). We intravenously administered 1.5e13 viral genomes per kilogram (vg/kg) AAV to adult mice via retro-orbital injections and harvested whole brains 6 weeks later (Fig. 4A). *Prnp* RT-qPCR and PrP ELISA on homogenized whole brain hemispheres revealed a 70–90% decrease in *Prnp* transcripts and a 60–80% reduction in PrP protein levels, with the protein knockdown possibly muted by ELISA floor effects (70). In addition, ZFcharm constructs lacking the KRAB domain were highly effective, suggesting DNA methylation alone is sufficient to silence *Prnp* in the brain (Fig. 4B). These levels of knockdown far exceed those previously achieved via ASO delivery shown to be protective against prion disease (6). Doubling the AAV dose led to a mild improvement in *Prnp* repression and reduced inter-individual variability (Fig. 4C). No adverse effects were detected at any of the administered doses, but subsequent experiments were conducted at the intermediate dose of 1.5e13 vg/kg given that the higher dose yielded modest gains. Nanopore sequencing of the 3 kb surrounding the *Prnp* promoter region showed that both ZFcharm and ZFcharm Kv1 established DNA methylation of CpGs surrounding the TSS (Fig. 4D and fig. S7). These data argue that CHARMs can recruit de novo DNA methyltransferases endogenously expressed in the brain and effectively release enzymatic autoinhibition in vivo.

We carried out in situ hybridization chain reaction (HCR) RNA-FISH on coronal brain sections to visualize *Prnp* expression six weeks post injection. Robust *Prnp* silencing was evident throughout the section, highlighting the broad CNS biodistribution attained by the AAV-PhP.eB capsid (Fig. 4E). Using HCR probes targeting the pan-neuronal marker *Uchl1*, we evaluated neuronal *Prnp* expression in ZFcharm Kv1-treated and untreated brains. While *Prnp* and *Uchl1* expression was colocalized throughout untreated brains, a decrease in *Prnp* signal was evident in most *Uchl1+* cells within treated brains (Fig. 4F). This effect was quantified at the single cell level using QuPath software (71), revealing that ZFcharm Kv1 effectively silenced *Prnp* in the vast majority of neurons, consistent with the data from whole brain hemisphere homogenate (Fig. 4G–I, Fig. 4B, and fig. S8A and B). This analysis establishes CHARM as a potent epigenetic silencer in the therapeutically-relevant cell type, as *Prnp* depletion in neurons alone is sufficient to prevent prion disease in mice (5, 72, 73). More broadly, it demonstrates that the CHARM technology is functional in post-mitotic cells.

### CHARMs can be programmed for time-limited expression through self-silencing.

AAV-mediated delivery of transgenes in non-dividing cells results in chronic expression from episomal AAV genomes, raising antigenicity and off-target editing concerns. As a result, previous efforts have aimed to restrict AAV expression once the desired therapeutic edits are accomplished (74–76). However, in the case of CRISPR cutting, this is at the cost of increased AAV transgene genomic insertions. Epi-editors are well-suited for a self-silencing approach as they do not induce DNA damage nor require constitutive expression to maintain target gene silencing.

**Fig. 5.**
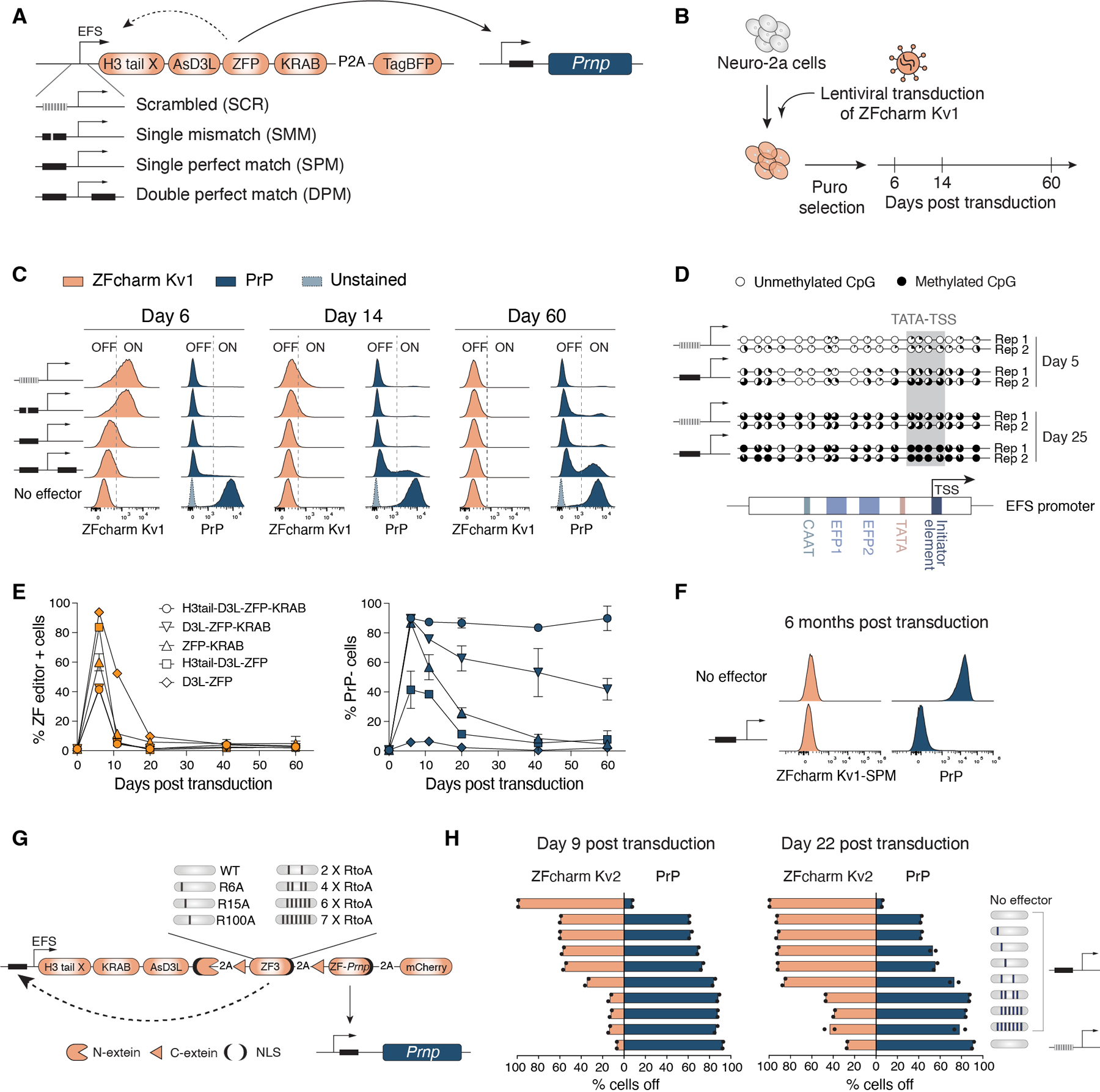
Transient CHARM expression through self-silencing is sufficient for persistent PrP repression. Self-silencing kinetics were quantified by measuring CHARM (orange; TagBFP or mCherry) and PrP (navy; Alexa Fluor 647 anti-PrP) expression over time via flow cytometry after lentiviral transduction of N2a cells. **(A)** Schematic of self-silencing CHARM constructs. **(B)** Schematic of experimental design. **(C)** Representative flow cytometry histograms of ZFcharm Kv1 and PrP expression at days 6, 14, and 60 post transduction. Dashed line indicates separation between expressing (‘ON’) and silenced (‘OFF’) cells. **(D)** Clonal bisulfite sequencing of EFS promoters driving ZFcharm Kv1-SCR and ZFcharm Kv1-SPM expression 5 and 25 days post transduction of N2a cells. % 5mCpG (black) across PCR clones is depicted as a pie chart. Sequence elements within the EFS promoter are shown in the schematic under the data. CpGs between the TATA box and TSS are highlighted in gray. **(E)** 60-day flow cytometry time course monitoring ZF editor and PrP expression across ZF-SPM constructs. Data are mean ± SD of n=2 replicates. **(F)** ZFcharm Kv1-SPM and PrP expression 6 months post transduction (n=1). **(G)** Schematic of experimental strategy to engineer a modular self-silencing ZFcharm Kv2 using two distinct ZF domains. **(H)** Tuning of self-silencing kinetics using an allelic series of ZF3 backbone RtoA mutations. ZFcharm Kv2 and PrP expression were quantified 9 and 22 days post transduction. Data are mean ± SD of n=2 replicates.

To achieve this, we developed self-silencing CHARMs, which silence their own promoter after silencing their target. We installed the ZFP binding motif from the *Prnp* promoter at positions flanking the core EF1ɑ (EFS) promoter driving CHARM expression, allowing the ZF domain to bind both the *Prnp* and EFS promoters (Fig. 5A). Self-silencing kinetics were assessed in N2a cells by measuring ZFcharm Kv1 and *Prnp* expression over time following lentiviral transduction with constructs containing the following binding site configurations: (1) a scrambled sequence as a negative control (scrambled; ZFcharm Kv1-SCR), (2) one binding site upstream of the promoter with a mismatch to decrease binding affinity (single mismatch; ZFcharm Kv1-SMM), (3) one binding site upstream of the promoter (single perfect match; ZFcharm Kv1-SPM), and (4) two binding sites flanking the promoter (double perfect match; ZFcharm Kv1-DPM) (Fig. 5, A and B).

Flow cytometry quantification after lentiviral transduction showed that all constructs initially induced complete repression of *Prnp* as well as differential rates of self-silencing, with only the SPM and DPM constructs showing self-silencing 6 days post-transduction (Fig. 5C). By 60 days post-transduction, ZFcharm Kv1 was fully silenced across all conditions, yet *Prnp* was reactivated in a subset of cells transduced with ZFcharm Kv1-DPM. Our interpretation is that the KRAB domain facilitated complete repression of *Prnp* initially, but self-silencing occurred too rapidly to establish lasting repression via DNA methylation (Fig. 5C). These findings are generalizable beyond lentiviral assays, as integrating the self-silencing constructs using the *piggyBac* transposase system produced consistent results (fig. S9A).

We selected ZFcharm Kv1-SPM for further characterization as it minimized the length of CHARM expression without compromising heritable silencing. Clonal bisulfite sequencing of the ZFcharm Kv1-SPM promoter in N2a cells revealed an accumulation of DNA methylation 5 days post transduction, particularly between the TATA box and the TSS (Fig. 5D and fig. S9, B and C). By day 25, this region was completely methylated (Fig. 5D and fig. S9, B and C). The promoter with a scrambled binding site also gained methylation over time, but in a slower and more dispersed fashion (Fig. 5D and fig. S9B). We attribute this gain in methylation and eventual loss of ZFcharm Kv1-SCR expression to self-silencing-independent transgene silencing (77).

To investigate the essentiality of each ZF-charm component, we compared ZFcharm Kv1-SPM to other ZF-SPM constructs lacking one or more domains. While all editors became self-silenced, only ZFcharm Kv1-SPM showed stable repression of *Prnp* over time (Fig. 5E and fig. S9D). Strikingly, *Prnp* remained transcriptionally silent 6 months post ZFcharm Kv1-SPM transduction in N2a cells (Fig. 5F). In contrast to our in vivo data, the KRAB domain was required for robust *Prnp* repression in the context of dividing cells in culture (Fig. 4B, Fig. 5E, and fig. S9D). Inclusion of the KRAB domain in CHARM constructs should therefore be determined on a locus-by-locus basis based on efficacy and off-target profiles.

We next engineered a more modular self-silencing ZFcharm Kv2 which eliminates the need to adjust self-silencing kinetics for each new target. To accomplish this, we integrated two ZF domains into our lentiviral construct, with one exclusively responsible for self-silencing and the other for target gene repression (Fig. 5G). To comply with the AAV packaging limit, we optimized the construct to incorporate the Npu intein strategy described above, where a C-terminal N-extein and an N-terminal C-extein are fused to the CHARM domains and each ZF, respectively (Fig. 5G and fig. S10, A to C). We selected a previously characterized synthetic ZF, ZF3, for the self-silencing component (66).

Placing a single ZF3 binding site upstream of the EFS promoter resulted in complete self-silencing and minimal *Prnp* repression. We slowed the kinetics by cloning an allelic series of arginine-to-alanine (RtoA) mutations in the ZF3 backbone, with the added benefit of reducing off-target interactions (40, 78–80). Introducing two RtoA mutations in the ZF3 backbone slowed self-silencing enough for ZF-*Prnp* to first establish heritable *Prnp* repression without abrogating self-silencing (Fig. 5H). We also tested point mutations in the ZF3 DNA binding site, illustrating an alternative method to decrease the rate of self-silencing (fig. S10, D and E). Together, these results highlight the potential of self-silencing CHARMs to constrain transgene expression when employing delivery modalities which result in sustained cargo expression.

### Self-silencing CHARMs are functional in vivo

**Fig. 6.**
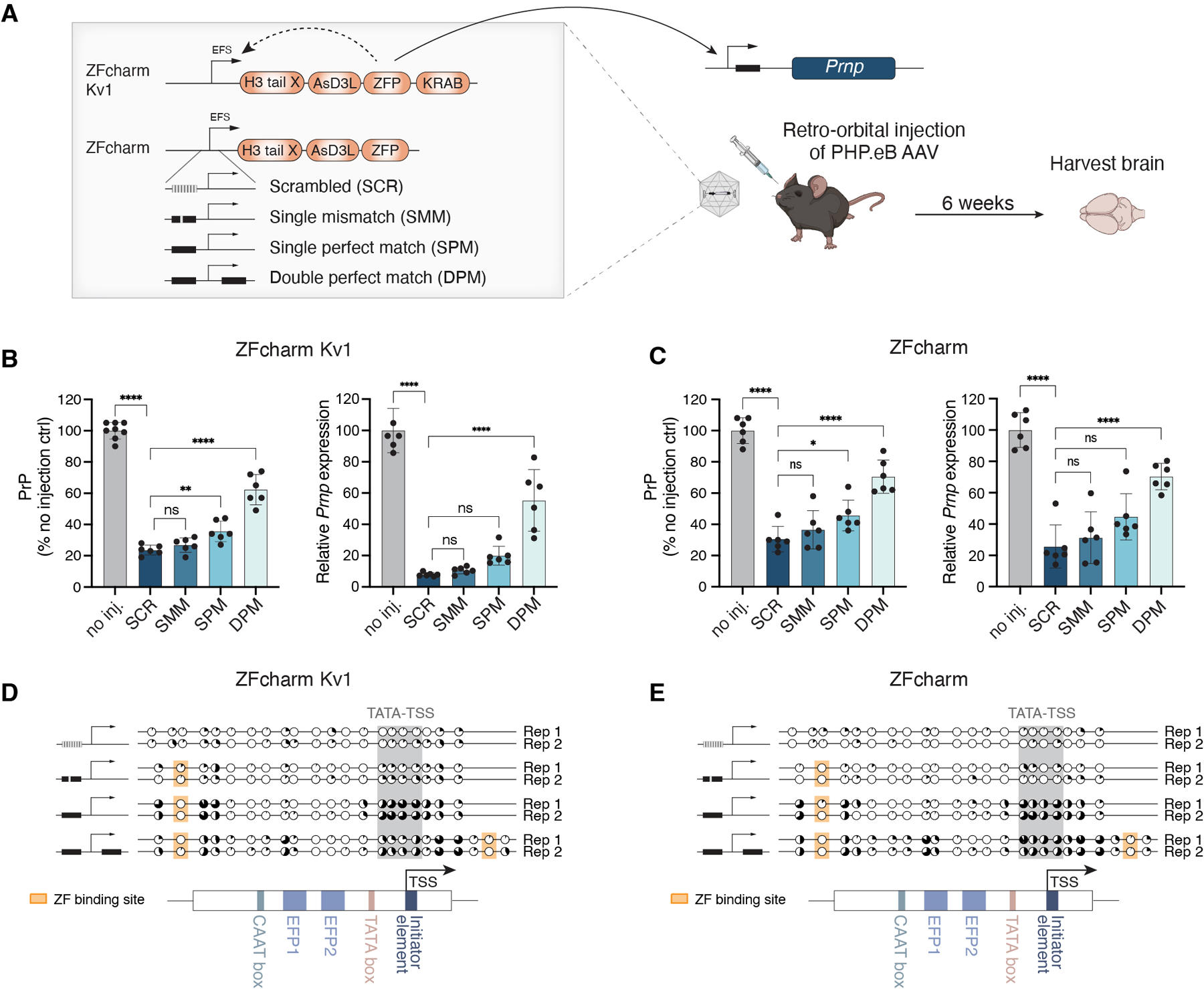
Self-silencing ZFcharm is functional in vivo. **(A)** Schematic of experimental design. **(B** and **C)** PrP ELISA and *Prnp* RT-qPCR data generated from brain hemisphere homogenate 6 weeks post injection of 1.5e13 vg/kg AAV. AAV capsids were packaged with self-silencing ZFcharm constructs containing **(B)** or lacking **(C)** the KRAB domain. Data are mean ± SD of n=6–8 replicates. Statistical analyses are one-way ANOVAs followed by Tukey’s multiple comparisons test (* p < 0.05; ** p < 0.005; *** p < 0.0005; **** p<0.0001; ns, not significant). **(D** and **E)** Clonal bisulfite sequencing of the EFS promoter driving expression of self-silencing ZFcharm constructs containing **(D)** or lacking **(E)** the KRAB domain. % 5mCpG (black) is calculated for each CpG site across PCR clones and depicted as a pie chart. Sequence elements within the EFS promoter are shown in the schematic under the data. CpGs between the TATA box and TSS are highlighted in gray and the ZF binding site is highlighted in orange.

We packaged AAV capsids with the same self-silencing CHARM constructs tested in vitro (ZFcharm Kv1 and ZFcharm with DPM, SPM, SMM, and SCR self-silencing binding sites) to assess whether the self-silencing approach would translate in vivo (Fig. 6A). *Prnp* expression in the brain was strongly reduced across all conditions 6 weeks post AAV injection, with an inverse relationship between the speed of self-silencing and the degree of *Prnp* knockdown (Fig. 6, B and C). The KRABless self-silencing ZFcharm constructs again yielded similar results, arguing that DNA methylation alone is effective in suppressing episomal AAV transgenes in addition to endogenous genes (Fig. 6C).

To confirm that self-silencing CHARMs methylate their own promoter in vivo, we carried out clonal bisulfite sequencing on episomal AAV DNA extracted from brain homogenate. The SPM and DPM promoters acquired DNA methylation at the CpGs surrounding the TSS and next to the ZF binding site, matching the pattern observed in cultured cells (Fig. 6, D and E, fig. S11, A and B, and Fig. 5D). By contrast, the EFS promoter was completely unmethylated in single-stranded AAV genomic DNA extracted from ZFcharm Kv1-SPM AAV particles, indicating that self-silencing occurred after brain transduction and not during viral packaging (fig. S11C).

To assess the durability of *Prnp* repression following self-silencing in vivo, we quantified *Prnp* expression and AAV promoter methylation 13 weeks post injection of ZFcharm Kv1-packaged AAV (fig. S12A). The relationship between self-silencing efficiency and *Prnp* knockdown persisted, with no evidence of *Prnp* reactivation (fig. S12, B and C). *Prnp* promoter methylation was consistent with these results (fig. S13, A and B), and DNA methylation of the TSS driving ZFcharm expression in the SPM and DPM conditions was also maintained (fig. S12D). Collectively, our findings indicate that CHARM can be engineered to silence itself in vivo after silencing its target. Additional optimization of self-silencing kinetics will further improve the balance between target repression and timely discontinuation of transgene expression, such as by modulating the number of CpG sites in the promoter (81). For instance, systems for small molecule control of self-silencing, such as tamoxifen-induced nuclear localization of a synthetic ZF transcriptional repressor (66), could allow for more precise temporal control as well as compatibility with different DNA-binding domains.

## Discussion

The promise of genetic medicines has been limited by the challenges of delivering the large and complex effector complexes (e.g. Cas9-sgRNA ribonucleoproteins) typically required to mediate permanent changes to the genome or epigenome (82) as well as toxicity and unintended consequences caused by repair of double stranded breaks and single stranded nicks. Here we present CHARM, a compact, programmable and readily deliverable DNA methylation system capable of permanently silencing targeted genes with high specificity. CHARM leverages the existing cellular machinery thus obviating the need to overexpress any catalytic domain. As such, these effectors are smaller and potentially less cytotoxic than existing technologies and do not rely on DNA sequence edits (83–90). Unlike genome editing approaches that disrupt coding regions or splice sites (91, 92), CHARM does not lead to the permanent production of an altered mRNA encoding for a truncated protein.

The CHARM system can be readily encoded within the genome of AAV vectors when coupled with ZFPs, TALEs, or small CRISPR-Cas DNA-binding domains. AAV-based delivery has been approved for indications in a variety of tissues including the CNS, muscle, and blood (93). ZFcharm represents the first AAV-delivered tool capable of gene silencing through targeted DNA methylation. Specifically, we show in mice that the CHARM system can establish stable DNA methylation and transcriptional silencing of the prion protein in the majority of neurons, a post-mitotic cell type, which argues for its utility in preventing other neurodegenerative diseases caused by a buildup of toxic protein aggregates (15, 16). The small size of ZFcharm enables a range of strategies for optimizing delivery and efficacy, which we illustrate by developing modular and tunable self-silencing ZFcharms. This can be extended to multiplexed targeting using up to three distinct ZFcharms or the use of different promoters or 3’UTRs that drive robust cell-type specific expression. The major components of ZFcharm are either derived from or closely related to human proteins, so it is expected to have reduced antigenic propensity especially in the context of time-limited expression. More in-depth in vivo toxicity and off-target analyses are critical next steps in developing CHARM as a therapeutic.

The dominant mechanism of FDA-approved drugs is through inhibition of a target protein (94). Thus, while major challenges remain, long-term and reversible gene silencing is potentially applicable to prevent or treat a range of pathological processes. Additionally, silencing enhancers (32) could enable cell type-specific tuning of gene expression, and the relatively wide targeting window (~1 kb) of epigenetic silencers facilitates the use of single nucleotide polymorphisms for allele-specific targeting. A wide variety of AAV capsid variants are in development with tropism for different tissues (95, 96), including a recently-described engineered AAV capsid that crosses the blood-brain barrier by binding the human transferrin receptor, making it a promising candidate for human CNS delivery (97). Beyond AAVs, the compact and single-component nature of ZFcharm could greatly facilitate other delivery platforms. For example, the short mRNA of ZFcharm could be delivered by engineered virus-like particles or lipid nanoparticles (LNP) (82, 98, 99) without the need for co-delivery of guide RNAs and difficult-to-produce long mRNAs. This flexibility enables further applications of CHARM as both a therapeutic and a tool for studying chromatin biology.

Prion disease, which is currently untreatable and rapidly fatal, represents a promising area for the initial clinical development of AAV-delivered ZFcharms. Animal studies provide a strong rationale for the therapeutic targeting of the prion protein. Even after onset of symptoms, moderate decreases of PrP expression in neurons is sufficient to halt and even reverse the disease process (5), while complete inhibition of PrP expression is well tolerated across several mammalian species (7–11). Our demonstration of greater than 80% brain-wide knockdown of PrP expression far exceeds the minimal knockdown required for a therapeutic effect— 50% knockdown by an ASO extended survival with five different prion strains, and as little as 21% knockdown delayed symptom onset (100). Finally, both the mouse and human *PRNP* genes can be readily and stably silenced, and homology between the *PRNP* promoter in humans and nonhuman primates could enable the design of cross-reactive ZFcharms for preclinical studies. Beyond the potential in treating prion disease, therapeutic targeting of *PRNP* will also provide practical experience on the benefits and unforeseen challenges of broader clinical applications of CHARM.

## Materials and Methods

### Cell culture and cell line generation

HEK293T (ATCC, CRL-3216) and Neuro-2a (N2a; ATCC, CCL-131) cells were cultured in Dulbecco’s Modified Eagle Medium (DMEM) supplemented with 10% fetal bovine serum (FBS), 100 units/mL streptomycin, 100 μg/ml penicillin, and 2 mM glutamine. Cells were passaged every 2 to 3 days using Trypsin-EDTA (0.25%). Cell lines were cultured at 37°C with 5% CO2.

The mScarlet-*CLTA* cell line was generated by knocking in a 5′ *mScarlet* tag at the *CLTA* locus. The sgRNA sequence targeting *CLTA* was ligated into pX458 (Addgene #48138) to generate the Cas9 + sgRNA plasmid. A double-cut HDR donor plasmid with the *mScarlet* tag sequence flanked by 800 bp homology arms was cloned from a pUC19 backgone (Addgene #50005) using NEBuilder HiFi DNA Assembly (New England BioLabs, E2621L). Knock-in efficiency was increased by flanking the donor sequence with sgRNA-PAM sequences used to target the *CLTA* locus to induce linearization post transfection (101). The HDR donor and Cas9 + sgRNA plasmids were co-transfected into HEK293T cells using TransIT-LT1 Transfection Reagent (Mirus Bio, 10767-122). mScarlet+ cells were sorted by FACS 6 days post transfection and successful tag insertion was validated via PCR.

### Plasmid design

Guide RNAs were designed using CRISPick SpCas9 CRISPRi guide prediction software (102). The sgRNA-expressing lentiviral vectors were constructed by ligation of annealed oligonucleotides (IDT) downstream of the mU6 promoter using BstXI and BlpI restriction sites. The vector also expresses HaloTag7 to allow for transfection and infection rate measurement by staining with Janelia Fluor HaloTag Ligands (Promega, GA1110). Cloning AAV plasmids and CHARM constructs was performed with eBlocks DNA fragments (IDT), oligonucleotides (IDT), or PCR amplicons produced from appropriate template sequences using Q5 Hot Start High-Fidelity 2x Master Mix (New England BioLabs, M0494L) or KOD Xtreme Hot Start DNA Polymerase (EMD Millipore, 719753). DNA fragments were cloned into restriction enzyme-digested plasmids using NEBuilder HiFi DNA Assembly (New England BioLabs, E2621L). All plasmids were sequence-confirmed by long-read whole plasmid sequencing by Quintara Bio. Optimized CHARM sequences can be found in Table S2.

### Plasmid transfection

Transient transfection experiments in N2a cells were performed in 6-well plates using TransIT-LT1 Transfection Reagent (Mirus Bio, 10767-122) and Opti-MEM Reduced Serum Medium (Thermo Fisher Scientific, 31985062). Cells at 70% confluency were transfected with 2.5 µg of plasmid. Cells co-transfected with plasmid encoding CRISPRoff or CRISPRi and plasmid encoding sgRNA were transfected with 1.7 µg and 800 ng, respectively. Transient transfection experiments in HEK293T cells were performed in 24-well plates using polyethylenimine (PEI). Cells at 70% confluency were transfected with 250 ng of plasmid. Transfected cells were sorted on TagBFP expression 2 days post transfection on a SONY MA900 and re-plated at a density of 120K cells/well in a 24-well plate. Cells were given four days to recover without changing media. Beginning at six days post-transfection, cells were assessed for fluorescence markers using the Attune NxT Flow Cytometer and passaged at a 1:8 dilution every two days for the duration of the time course.

### Lentiviral packaging and transduction

Lentiviral particles were produced by co-transfecting lentiviral transfer plasmids with standard packaging vectors psPAX2 (Addgene #12260) and pMD2.G (Addgene #12259) into HEK293T using FuGENE HD (Promega, PAE2311) or PEI. Media was replaced with fresh media supplemented with ViralBoost (Alstem, NC0966705) 6 hours post-transfection. Viral supernatants were harvested 48 hours after transfection and flash-frozen. Lentiviral transductions were performed in polybrene-supplemented media (8 μg/ml). Media was replaced the following day and selection with 2 µg/mL puromycin was initiated two days post transduction.

### PiggyBac transfection

The Super PiggyBac Transposase Expression Vector (System Biosciences, PB210PA-1) and CHARM-expressing *PiggyBac* transposon vector were co-transfected at a 1:10 molar ratio into N2a cells using TransIT-LT1 Transfection Reagent (Mirus Bio, 10767-122). Selection with 2 µg/mL puromycin was initiated 2 days post transfection. Cells were assessed for ZFcharm Kv1 and PrP expression using immunofluorescence staining (see below) followed by flow cytometry using the Attune NxT Flow Cytometer.

### Immunofluorescence staining

Staining for cell surface proteins PrP, CD51, CD81, and CD151 was performed on cells at 50–90% confluency in 24-well plates. Cells were resuspended in PBS using mechanical force and transferred to a 96-well V-bottom plate. Cells were incubated at 4°C in the dark for 30 minutes with the appropriate fluorophore-conjugated antibody (Alexa Fluor 647 6D11 anti-PrP, also called anti-CD230, Biolegend, 808007; APC anti-human CD81, Biolegend, 349509; APC anti-human CD55, Biolegend, 311311; APC anti-human CD151, Biolegend, 350405) at a concentration of 0.5ug/mL. Cells were washed twice in PBS supplemented with 5% FBS and read out on the Attune NxT Flow Cytometer.

### Cell viability staining

To assess cytotoxicity of the different epi-editors, HEK293T cells were transiently transfected with ZFP expressing constructs followed by FACS on TagBFP expression two days later. After recovering from FACS for four days, 1e6 cells were trypsinized, spun down at 400xg for 5 minutes, and resuspended in 1 mL of PBS. One μL of LIVE/DEAD^™^ Fixable Near-IR Dead Cell Stain for 633 or 635 nm excitation (Invitrogen^™^ L34975) dissolved in DMSO was added to the cells and kept on ice for 30 minutes protected from light. Cells were pelleted and washed with PBS twice followed by resuspension in 150 μL of PBS and flow cytometry on the Attune NxT Flow Cytometer. Total viable cells per 100 μL were counted based on near-IR (~780 nm) fluorescence.

### Generation of genetic knockout cell lines

To knock out *DNMT3A* and *DNMT3B* in our mScarlet-CLTA reporter HEK293T cell line, we nucleofected Alt-R^™^ S.p. HiFi Cas9 Nuclease V3, 100 µg (IDT 1081060) complexed with guide RNA to form ribonucleoprotein (RNP). Guide RNAs designed using CRISPick software (102) were formed by mixing 5 µL Alt-R^®^ CRISPR-Cas9 crRNA at 100 µM (IDT custom designs; Table S1) and 5 µL Alt-R^®^ CRISPR-Cas9 tracrRNA at 100 µM (IDT 1072533) and boiling at 95 C for 5 minutes followed by cooling to room temperature. To make RNP, 1.4 µL of the 50 µM annealed guide RNA was mixed with 1 µL of the Cas9 nuclease (62 µM) and 0.6 µL of phosphate buffered saline to a final volume of 3 µL, which was incubated at room temperature for 20 minutes and then placed on ice until ready for electroporation. HEK293T reporter cells were dissociated with trypsin and 1e6 cells were spun down. These were resuspended in Amaxa^™^ SF cell line 4D-nucleofector buffer with Cas9 RNP added and nucleofected in a 100 µL cuvette using program CM-130 following manufacturer’s protocols (V4XC-2024, Lonza). Cells were immediately plated in a 6-well dish containing pre-warmed media (see culture conditions above). Knockout efficiency was determined by seeding cells in a 96-well plate and lysing with 200 µL QuickExtract DNA Extraction Solution (SS000035-D2, Biosearch Technologies) following manufacturer protocols. Two µL of cell lysate was used as a template for a 40 µL PCR using 2x Super Pfx Mastermix (CW2965, Cowin Biosciences) and the following primers: CAGCCAGGCTCCTAGACCCA (*DNMT3A*, Fwd), GGCGGGGTCATGTCTTCAGG (*DNMT3A*, Rev), TGGCAGGAAAAACCCCGTGT (*DNMT3B*, Fwd), and AGCCGTTCCCTATACATGAGTTCT (*DNMT3B*, Rev) (5’ to 3’) to generate a 715 bp amplicon for *DNMT3A* and a 700 bp amplicon for *DNMT3B*. PCRs were purified using QIAquick PCR Purification Kit (28104, Qiagen) and Sanger sequenced by Quintara Bio. Insertion/deletion (indel) frequencies were determined from Sanger traces using Synthego ICE analysis (https://ice.synthego.com/#/).

### DNMT3L phylogeny construction

Genome-mining for DNMT3L orthologs and ancestral reconstructions was performed based on previously established methods (61). A list of ~200 *DNMT3L* orthologs was obtained by performing a BLASTP (103) search in the NCBI non-redundant protein sequences database, using the human and mouse DNMT3L amino acid sequences as a query, and removing sequences with >97% pairwise identity. A MAFFT multiple sequence alignment was performed using the FFT-NS-i (standard) strategy with a maximum of two iterations (104) and then used for phylogenetic tree construction implementing IQ-TREE software (105). With IQ-TREE we inferred the phylogenetic tree using the predicted best-fit model and ultrafast bootstrapping with 1000 replicates and optimized parameters. After visualization of the tree using the interactive tree of life (iTOL) v5 online tool (106), selected ancestral nodes were predicted with the IQ-TREE ASR function (105). Two dozen GenScript codon-optimized orthologs and ASRs were synthesized as DNA eBlocks (IDT). D3L sequences can be found in Table S2.

### TALE Design

TALE DNA-binding domains were constructed following published guidelines (107, 108). In brief, potential 18-nucleotide binding sites beginning with the invariable thymine were compiled from the mouse and human *PRNP* promoter regions and scored for specificity using nucleotide BLAST (103). Top candidates were selected for synthesis in the chimerized TALE scaffold (63) using the following repeat variable diresidues (RVDs): HD for cytosine, NG for thymine, NI for adenine, NH for guanine, and G* for any possible 5-methyl-cytosine within a CpG dinucleotide. Each TALE was synthesized as eBlocks (IDT) in two halves which were cloned into a CHARM acceptor vector using NEBuilder HiFi DNA Assembly (New England BioLabs, E2621L). TALE sequences can be found in Table S2.

### Extraction of HMW gDNA

To extract high molecular weight (HMW) genomic DNA (gDNA) from cells for Nanopore long-read sequencing analysis of CpG methylation, 1e^6^ cells were pelleted at 400xg for 5 minutes, rinsed with PBS, and pelleted again. Pellets were processed using the Monarch^®^ HMW DNA Extraction Kit for Cells & Blood (New England Biolabs, T3050L). To extract HMW gDNA from mouse brain tissue, two 150 μm coronal sections were cut from flash-frozen hemispheres embedded in optimal cutting temperature (O.C.T.) compound (see below) and collected in a single 1.5 mL Eppendorf tube. These were frozen at −80°C until ready for preparation. Prior to processing using the Monarch^®^ HMW DNA Extraction Kit for Tissue (New England Biolabs, T3060L), these sections were rinsed with ice-cold PBS twice and pelleted on a tabletop microcentrifuge (MyFuge 12 Mini Centrifuge, Benchmark Scientific C1012) to remove excess O.C.T. The gDNA extraction was performed following manufacturer instructions with slight modifications to maximize yield; three glass beads were used instead of two, and gDNA was eluted in 200 μL of water heated to 65°C. To concentrate the gDNA for Nanopore library preparation (to ~5 μg DNA in <24 μL), gDNA in the eluate was precipitated by adding 2 μL 20 mg/mL glycogen (Thermo Scientific, R0561), 22 μL 3M pH 5.2 sodium acetate, and 155 μL pure room temperature isopropanol followed by mixing and centrifugation at 15,000xg for 20 minutes at 4°C. Supernatant was carefully decanted and DNA pellets were washed with 1 mL 70% ethanol and centrifuged at 15,000xg again for 10 minutes at 4°C. Supernatant was decanted and the pellet was air-dried for 10 minutes. The DNA pellet was redissolved in 25 μL water at 56°C for two hours. Wide-bore pipette tips (Genesee Scientific, 22–427 and 22–424) were used for all gDNA handling steps to prevent shearing.

### Target enrichment and Nanopore library preparation

Two upstream and two downstream guide RNAs were designed flanking the *PRNP* locus in a ~5 kb window using CHOPCHOPv3 (109). Alt-R^®^ CRISPR-Cas9 tracrRNA (IDT, 1072533) and custom Alt-R^®^ CRISPR-Cas9 crRNA (IDT) were annealed at 10 μM in nuclease-free duplex buffer (IDT, 11-01-03-01). In a 1.5 mL Eppendorf tube, 79.2 μL of water was combined with 10 μL of reaction buffer (RB) from the Cas9 Sequence Kit Cas9 Sequencing Kit (Oxford Nanopore Technologies, SQK-CS9109), 10 μL of 10 μM pooled annealed guide RNAs, and 0.8 μL of 62 μM Cas9 nuclease (Alt-R^™^ S.p. HiFi Cas9 Nuclease V3, IDT 1081060) and was complexed at room temperature for 30 minutes before use. Prior to Nanopore sequencing of native DNA molecules, the prion locus was enriched using 5 μg of input gDNA and prepared for sequencing following manufacturer’s protocols (ONT, SQK-CS9109).

### Nanopore sequencing

Target-enriched Nanopore libraries were loaded into a MinION Flow Cell R9.4.1 (ONT, FLO-MIN106D) after priming with the flow cell priming kit (ONT, EXP-FLP002) and sequenced on a MinION sequencing device (ONT, MIN-101B) with MinKNOW software (ONT, v.23.04.6) using fast base calling combined with adaptive sampling to further enrich the target locus. Live base calling was enabled by hardware (13th Gen Intel Core i7-13700 2.10 GHz and 16 GB RAM) with a GPU (NVIDIA, GeForce RTX 3070) running Windows 11 Pro. Each library was sequenced in series, and the flow cell was washed using the flow cell wash kit (ONT, EXP-WSH004) between samples. Each flow cell could run 3–4 libraries before requiring replacement. Two biological replicates were sequenced for each sample type.

### Nanopore base calling and data analysis

Base calling was performed on the raw FAST5 files with Guppy (ONT, v.6.5.7), using a configuration file for high-accuracy modified DNA base calling on an R9.4.1 pore at 450 bases s^−1^. The resulting reads were then mapped to the GRCh38 (human) or GRCm39 (mouse) reference genome without alternate contigs using minimap2 v.2.26 with default settings for alignment of nanopore reads (-x map-ont). Reads were filtered based on reciprocal 90% coverage with the target locus using the bedtools v.2.31.0 intersect (-wo -f 0.9 -r) command. Filtered, sorted, and indexed bam output files were used for methylation visualization (see below) or further processed using modkit tools (ONT, https://github.com/nanoporetech/modkit) and custom python scripts implementing Numpy v.1.26.3, Pandas v.2.2.0, and Seaborn v.0.13.2 for Pearson correlation and average methylation plots. Virtual environment files and custom scripts can be found here: https://github.com/edwin-n-neumann/CHARM_x_Prion.

### Visualization of DNA methylation on individual Nanopore reads

Output bam files with read names numbered by average methylation were indexed and loaded into Integrative Genomics Viewer (110) v2.16.2 with the following settings: squished, small indel threshold <100 bp, hide mismatched bases, hide insertion markers, quick consensus mode, color by 5mC, sort by Read Name, Reverse Sort. To change colors to black (unmethylated) and blue (methylated), exported PNG files were adjusted with an Adobe Photoshop 2024 batch processing script for consistency.

### RNA-seq analysis

N2a cells were maintained for 28 days post lentiviral transduction of ZFcharm Kv1 or CRISPRcharm Kv1 constructs. CRISPRcharm Kv1 was introduced into cells already expressing either a non-targeting sgRNA or a sgRNA targeting *Prnp.* An empty lentiviral vector was used as a no-editor control. Each transduction was done in triplicate. Cells were dislodged from 6-well plates using Trizol and total RNA was extracted using the Direct-zol RNA Miniprep Kit (Zymo, R2051). Libraries were prepared using the KAPA RNA HyperPrep Kit with RiboErase (HMR) (Roche, KK8560) and sequenced as 50 bp single-end reads on a NovaSeq 6000 (Illumina). Raw sequencing reads were aligned to the mouse genome (mm39) using STAR 2.7.1a and quantified using featureCounts 1.6.2 (111). Differential expression analysis was carried out using DESeq2 (112) using default parameters. The lfcShrink function was applied using the apeglm shrinkage estimator. Sequencing data are available on GEO (GSE255987).

### Clonal bisulfite sequencing

Clonal bisulfite sequencing (32) of the EFS promoter was performed on (1) genomic DNA extracted from lentivirally transduced N2a cells, (2) double-stranded AAV genomes extracted from brain homogenate, or (3) single-stranded AAV genomes extracted from viral particles. N2a genomic DNA was extracted using the PureLink Genomic DNA Mini Kit (Invitrogen, K182001). AAV episomal DNA was obtained via Trizol-Chloroform extraction from brain homogenate followed by treatment with T5 exonuclease (New England BioLabs, M0663S) and RNase Cocktail Enzyme Mix (Thermo Fisher Scientific, AM2288). To extract single-stranded AAV DNA, viral particles were treated with Turbonuclease (MilliporeSigma, T4330) to digest contaminating plasmid DNA and then with Proteinase K to digest viral capsids. Both double- and single-stranded AAV DNA was purified with the DNA Clean & Concentrator-5 Kit (Zymo, 11-302B). Bisulfite conversion was performed on 100–500 ng DNA using the EZ DNA Methylation Lightning Kit (Zymo, D5001). Purified bisulfite-converted DNA was amplified with forward primer GAGTGGTTAATTTTATTATTAGGGGT (5′ to 3′) and reverse primer TTTCTAACAATTTATTTAATCCTAACCA (5′ to 3′) using EpiMark Hot Start Taq (New England BioLabs, M0490S), and purified using a QIAquick PCR Purification Kit (QIAGEN, 28104). Amplicons were cloned into pCR2.1-TOPO Vector using a TOPO TA Cloning Kit (Invitrogen, 451641) and transformed into Stellar Competent E. coli Cells (Takara Bio, 636766). Cells were plated on plates supplemented with carbenicillin, X-gal, and IPTG for blue-white screening. Colonies were sequenced by Sanger sequencing and reads were processed for display using QUMA software (114).

### AAV production and titering

Recombinant AAVs (AAV-PHP.eB) were produced in suspension HEK293T cells, using F17 media (Thermofisher, A138501). Cell suspensions were incubated at 37°C, 8% CO_2_, 80 RPM. 24 hours before transfection, cells were seeded in 500–1000 mL at ~1 million cells/mL. The day after, cells (~2 million cells/mL) were transfected with pHelper, pRepCap, and pTransgene (2:1:1 ratio, 2 µg total DNA per million cells) using Transport 5 transfection reagent (Polysciences, 26008–50) with a 2:1 PEI:DNA ratio. Three days post-transfection, cells were pelleted at 2000 RPM for 12 minutes into Nalgene conical bottles. The supernatant was discarded, and cell pellets were stored at −20°C until purification. Each pellet, corresponding to 500 mL of cell culture, was resuspended in 14 mL of 500 mM NaCl, 40 mM Tris-base, 10 mM MgCl_2_, with Salt Active Nuclease (ArcticZymes, #70920-202) at 100 U/mL. Afterwards, the lysate was clarified at 5000 RCF for 20 minutes and loaded onto a density step gradient containing OptiPrep (Cosmo Bio, AXS-1114542) at 60%, 40%, 25%, and 15% at a volume of 6, 6, 8, and 5 mL, respectively, in OptiSeal tubes (Beckman, 342414). The step gradients were spun in a Beckman Type 70ti rotor (Beckman, 337922) in a Sorvall WX+ ultracentrifuge (Thermo Scientific, 75000090) at 67,000 RPM for 75 minutes at 18°C. Afterwards, ~4.5 mL of the 40–60% interface was extracted using a 16-gauge needle, filtered through a 0.22 μm PES filter, buffer exchanged with 100K MWCO protein concentrators (Thermo Scientific, 88532) into PBS containing 0.001% Pluronic F-68, and concentrated down to a volume of 200–1000 μL. The concentrated virus was filtered through a 0.22 μm PES filter and stored at 4°C or −80°C.

To determine AAV titers, 5 μL of each purified virus library was incubated with 100 μL of an endonuclease cocktail consisting of 1000U/mL Turbonuclease (Sigma T4330-50KU) with 1X DNase I reaction buffer (New England BioLabs, B0303S) in UltraPure DNase/RNase-Free distilled water at 37°C for one hour. Next, the endonuclease solution was inactivated by adding 5 μL of 0.5 M EDTA, pH 8.0 (ThermoFisher Scientific, 15575020) and incubated at room temperature for 5 minutes and then at 70°C for 10 minutes. To release the encapsidated AAV genomes, 120 μL of a Proteinase K cocktail consisting of 1 M NaCl, 1% N-lauroylsarcosine, 100 μg/mL Proteinase K (QIAGEN, 19131) in UltraPure DNase/RNase-Free distilled water was added to the mixture and incubated at 56°C for 2–16 hours. The Proteinase K-treated samples were then heat-inactivated at 95°C for 10 minutes. The released AAV genomes were serial diluted between 460–4,600,000X in dilution buffer consisting of 10X PCR Buffer (Thermo Fisher Scientific, N8080129), 2 μg/mL sheared salmon sperm DNA (Thermo Fisher Scientific, AM9680), and 0.05% Pluronic F68 (Thermo Fisher Scientific, 24040032) in UltraPure Water (Thermo Fisher Scientific). 2 μL of the diluted samples were used as input in a ddPCR supermix (Bio-Rad, 1863023). Primers and probes, targeting the ITR region, were used for titration at a final concentration of 900 nM and 250 nM (ITR2_Forward: 5’-GGAACCCCTAGTGATGGAGTT-3’; ITR2_Reverse: 5’-CGGCCTCAGTGAGCGA-3’). The droplets were transferred to the thermocycler and cycled according to the manufacturer's protocol with an annealing/extension of 58°C for one minute. Finally, droplets were read on a QX100 Droplet Digital System to determine titers.

### Mice

All in vivo experiments were approved by the Institutional Animal Care and Use Committee of the Broad Institute (Protocol #0162-05-16-2, most recent approval date: 2024-01-03) and were performed in accordance with the National Institutes of Health *Guide for the Care and Use of Laboratory Animals*. Experiments in this study used 192 C57BL/6N mice (90 female, 102 male) obtained from Charles River Laboratories. Unless otherwise noted, mice were between 5–8 weeks old at the time of AAV injections.

### Intravenous AAV injection

Mice were anesthetized using inhaled isoflurane at 1–3%. AAV vectors (0.75e13, 1.5e13, or 3e13 vg/kg, ~100ml injection volume) were administered intravenously into the right retro-orbital sinus of the animal using a 300 μL insulin syringe with a 31G needle (328438, Becton Dickinson, USA) following established protocols (64, 113). One drop of 0.5% proparacaine (07-892-9554, Patterson Veterinary, USA) was applied topically to the eye immediately following injection. Mice were euthanized using CO_2_ inhalation at timepoints of 6- or 13-weeks post-injection, following which the brains were harvested and cut in half. One hemisphere was placed in a microtube and flash-frozen on dry ice for ELISA (see below), while the other hemisphere was prepared for histological analysis. In brief, a small amount of optimal cutting temperature (OCT) compound (Tissue-Tek 4583, Sakura, USA) was placed into a 15x15x5 mm cryomold (Tissue-Tek 4566, Sakura, USA), the hemisphere was placed cut side down into the mold, and fully covered with additional OCT compound prior to being flash-frozen on dry ice. All samples were stored at −80°C until further processing.

### Mouse perfusions

Mice were deeply anesthetized under 2–5% isoflurane and 0.5–1 LPM oxygen in an induction chamber. Mice were then transferred to a nose cone providing 2–5% isoflurane and 0.5–1 LPM oxygen. Anesthesia depth was validated with lack of bilateral toe pinch prior to the start of the surgical procedure. Mice were continuously monitored throughout the procedure for any signs of responsiveness. Paw color and respiration rate were monitored at all times during anesthesia. Once anesthesia was stable and at an acceptable plane for surgery (based on lack of a toe-pinch and eye blink response, and stable slow respiratory rate), an incision was made through the skin below the ribcage and blunt dissection scissors were used to separate the outer layers of skin from the cavity wall. A mid-sternal thoracotomy was then performed to expose the heart and great vessels. Perfusate was delivered using a needle through the left ventricle and an incision was made in the right atrium to provide an outflow for blood and perfused fluids.

Perfusion was carried out with ice-cold saline solution followed by phosphate buffered saline containing 4% paraformaldehdye (PFA). Perfusion was complete when outflow perfusate showed no visual trace of blood, and the animal had no cardiac or respiratory activity. Mice were decapitated prior to brain dissection.

### Brain homogenization

One hemisphere was homogenized at 10% wt/vol in cold 0.2% CHAPS solution prepared in 1X PBS with 1 tablet protease inhibitor (Roche cOmplete 4693159001, Millipore Sigma, USA) per 10 mL in 7 mL tubes pre-loaded with zirconium oxide beads (Precellys, Bertin, USA), using 3 x 40 second pulses on a Bertin MiniLysis Homogenizer (Bertin, USA). Homogenate was aliquoted into 40 μL aliquots for protein analysis and 300 μL aliquots for qPCR analysis, and stored at −80°C until further analysis.

### Protein analysis

PrP concentration in the brain was quantified using a previously published PrP ELISA (70). Briefly, the assay uses EP1802Y antibody (ab52604, Abcam, USA) for capture and biotinylated 8H4 antibody (ab61409, Abcam, USA) for detection, with streptavidin-HRP (Pierce High Sensitivity, 21130, Thermo Fisher Scientific, USA) and TMB substrate (7004P4, Cell Signaling Technology, USA). Recombinant mouse PrP (MoPrP23-231) prepared as described (115) was used for a standard curve. Protein knockdown was calculated by dividing the concentration of residual PrP in each treatment brain, by the mean concentration of residual PrP in the saline control brains from the same time point.

### RT-qPCR

Mouse *Prnp* RNA was quantified using RT-qPCR. RNA extracts were treated with DNase I (New England BioLabs, M0303S). Library preparation was performed using the RevertAid First Strand Synthesis Kit (Thermo Fisher Scientific, K1691). Taqman qPCR (Thermo Fisher Scientific, 4331182) was performed on cDNA samples using the QuantStudio 7 Flex (Applied Biosystems). ΔΔCt values were calculated based on the amplification of *Gapdh*, and normalized to the mean of the no injection controls. Probe and quencher sequences were purchased from Fisher Scientific as premixed Gene Expression Assays (*Gapdh* control, ID Mm99999915_g1; *Prnp* target, ID Mm07296968_m1).

### Tissue processing and sectioning

Whole mouse brains harvested from perfused mice were incubated overnight at 4°C in 4% PFA. Fixed brains were then washed in 1X PBS and dehydrated overnight at 4°C in 30% sucrose, followed by a second overnight incubation at 4°C in a 1:1 mixture of 30% sucrose and O.C.T. compound (Tissue-Tek, 4583). Dehydrated brains were placed in cryomolds containing O.C.T. and snap-frozen in liquid nitrogen-chilled isopentane. 10 µm coronal brain sections were cut using a Leica CM3050 S Research Cryostat and placed on SuperFrost Plus slides (VWR, 48311-703). Brains used to extract DNA for Nanopore long-read sequencing were harvested from non-perfused mice and directly embedded in O.C.T. before freezing on dry ice. These were cut into 150 μm sections using a Leica CM3050 S Research Cryostat and stored in tubes at −80°C before use.

### Hybridization chain reaction RNA fluorescence in situ hybridization (HCR RNA-FISH)

Coronal brain sections on SuperFrost Plus slides were immersed in 4% PFA at 4°C for 15 minutes and then sequentially immersed in 50% ethanol, 70% ethanol, 100% ethanol, and 1X PBS at room temperature for 5 minutes. A hydrophobic barrier was drawn around the tissue using an ImmEdge^™^ Hydrophobic Barrier Pen (Vector Laboratories, 101098-065). Third-generation multiplexed HCR RNA-FISH was performed as previously described (116). Briefly, tissue samples were pre-hybridized in hybridization buffer (Molecular Instruments) at 37°C for 10 minutes and then incubated in a 37°C humidified chamber overnight with split-initiator probes hybridizing to the *Prnp* and *Uchl1* mRNA transcripts diluted to a concentration of 4 nM in Hybridization Buffer. Split-initiator probes were purchased from Molecular Technologies. The slides were then immersed in 75%, 50%, and 25% probe wash buffer (Molecular Instruments) solutions at 37°C for 15 minutes, followed by two incubations in 5X SSCT, one for 15 minutes at 37°C and another for 5 minutes at room temperature. Tissue sections were then equilibrated in amplification buffer (Molecular Instruments) for 30 minutes at room temperature. Separately, metastable fluorescent hairpins conjugated to Alexa Fluor 647 and Alexa Fluor 546 were snap-cooled and diluted to 60 nM in amplification buffer. Samples were incubated in hairpin solution overnight in a dark humidified chamber at room temperature. Excess hairpin amplifiers were removed the next day in 5X SSCT at room temperature before staining with 1 µg/mL DAPI for 10 minutes, washing again in 5X SSCT, and mounting in VECTASHIELD^®^ PLUS Antifade Mounting Medium (Vector Laboratories, H-1900). Brain sections were imaged as z-stack tile scans on a Zeiss LSM 980 with Airyscan 2 Laser Scanning Confocal with a 20X objective.

### Image analysis

Maximum orthogonal projections and stitching of z-stack tile scales was performed using ZEN Blue software (Zeiss). Cell detection and classification was carried out using QuPath software v0.5.0 (71). Briefly, cells were detected using QuPath’s cell detection tool on the DAPI channel (cell expansion = 4 µm). QuPath’s built-in machine learning classification tool was used to detect neurons (using *Uchl1*-Alexa Fluor 647 signal) and *Prnp*+ cells (using *Prnp*-Alexa Fluor 546 signal). Multiple images were used to train the classifiers. Zoomed-in images of brain regions were median filtered using Fiji software v2.9.0 (117).

### Statistical analyses

All statistical tests performed in this study are indicated in the figure legends.

## Supplementary Material

Supplement

Table S2

MDAR Checklist

## Data Availability

RNA-seq datasets are available on GEO (GSE255987). Expression plasmids will be available from Addgene under a uniform biological material transfer agreement. All other data are available in the manuscript or the supplementary materials.
