# Supplementary material for: Brain-wide silencing of prion protein by AAV-mediated delivery of an engineered compact epigenetic editor
Source: Science. Author manuscript; Available in PMC 2025 Mar 3. (PMC11875203; doi:10.1126/science.ado7082)
Supplement: Supplement [file NIHMS2046549-supplement-Supplement.pdf]

Science
AAAS

Supplementary Materials for

Brain-wide silencing of prion protein by AAV-mediated delivery of an engineered compact epigenetic editor

Authors: Edwin N. Neumann[1,2]†, Tessa M. Bertozzi[1,3]†, Elaine Wu[1], Fiona Serack[4], John W. Harvey[4,] Pamela P. Brauer[4], Catherine P. Pirtle[4], Alissa Coffey[4], Michael Howard[5], Nikita Kamath[4], Kenney Lenz[5], Kenia Guzman[5], Michael H. Raymond[6,7], Ahmad S. Khalil[6,7,8], Benjamin E. Deverman[4], Eric Vallabh Minikel[4,9], Sonia M. Vallabh[4,9]*, Jonathan S. Weissman[1,3,10, 11]*

*Corresponding author. Email: weissman@wi.mit.edu (J.S.W.); svallabh@broadinstitute.org (S.M.V.)

The PDF file includes:

Materials and Methods
Figs. S1 to S13
Table S1

Materials and Methods

Cell culture and cell line generation
HEK293T (ATCC, CRL-3216) and Neuro-2a (N2a; ATCC, CCL-131) cells were cultured in Dulbecco's Modified Eagle Medium (DMEM) supplemented with 10% fetal bovine serum (FBS), 100 units/mL streptomycin, 100 µg/ml penicillin, and 2 mM glutamine. Cells were passaged every 2 to 3 days using Trypsin-EDTA (0.25%). Cell lines were cultured at 37°C with 5% CO2.

The mScarlet-*CLTA* cell line was generated by knocking in a 5′ *mScarlet* tag at the *CLTA* locus. The sgRNA sequence targeting *CLTA* was ligated into pX458 (Addgene #48138) to generate the Cas9 + sgRNA plasmid. A double-cut HDR donor plasmid with the *mScarlet* tag sequence flanked by 800 bp homology arms was cloned from a pUC19 backgone (Addgene #50005) using NEBuilder HiFi DNA Assembly (New England BioLabs, E2621L). Knock-in efficiency was increased by flanking the donor sequence with sgRNA-PAM sequences used to target the *CLTA* locus to induce linearization post transfection (*101*). The HDR donor and Cas9 + sgRNA plasmids were co-transfected into HEK293T cells using TransIT-LT1 Transfection Reagent (Mirus Bio, 10767-122). mScarlet+ cells were sorted by FACS 6 days post transfection and successful tag insertion was validated via PCR.

Plasmid design
Guide RNAs were designed using CRISPick SpCas9 CRISPRi guide prediction software (*102*). The sgRNA-expressing lentiviral vectors were constructed by ligation of annealed oligonucleotides (IDT) downstream of the mU6 promoter using BstXI and BlpI restriction sites. The vector also expresses HaloTag7 to allow for transfection and infection rate measurement by staining with Janelia Fluor HaloTag Ligands (Promega, GA1110). Cloning AAV plasmids and CHARM constructs was performed with eBlocks DNA fragments (IDT), oligonucleotides (IDT), or PCR amplicons produced from appropriate template sequences using Q5 Hot Start High-Fidelity 2x Master Mix (New England BioLabs, M0494L) or KOD Xtreme Hot Start DNA Polymerase (EMD Millipore, 719753). DNA fragments were cloned into restriction enzyme-digested plasmids using NEBuilder HiFi DNA Assembly (New England BioLabs, E2621L). All plasmids were sequence-confirmed by long-read whole plasmid sequencing by Quintara Bio. Optimized CHARM sequences can be found in Table S2.

Plasmid transfection
Transient transfection experiments in N2a cells were performed in 6-well plates using TransIT-LT1 Transfection Reagent (Mirus Bio, 10767-122) and Opti-MEM Reduced Serum Medium (Thermo Fisher Scientific, 31985062). Cells at 70% confluency were transfected with 2.5 µg of plasmid. Cells co-transfected with plasmid encoding CRISPRoff or CRISPRi and plasmid encoding sgRNA were transfected with 1.7 µg and 800 ng, respectively. Transient transfection experiments in HEK293T cells were performed in 24-well plates using polyethylenimine (PEI). Cells at 70% confluency were transfected with 250 ng of plasmid. Transfected cells were sorted on TagBFP expression 2 days post transfection on a SONY MA900 and re-plated at a density of 120K cells/well in a 24-well plate. Cells were given four days to recover without changing media. Beginning at six days post-transfection, cells were assessed for

fluorescence markers using the Attune NxT Flow Cytometer and passaged at a 1:8 dilution every two days for the duration of the time course.

Lentiviral packaging and transduction
Lentiviral particles were produced by co-transfecting lentiviral transfer plasmids with standard packaging vectors psPAX2 (Addgene #12260) and pMD2.G (Addgene #12259) into HEK293T using FuGENE HD (Promega, PAE2311) or PEI. Media was replaced with fresh media supplemented with ViralBoost (Alstem, NC0966705) 6 hours post-transfection. Viral supernatants were harvested 48 hours after transfection and flash-frozen. Lentiviral transductions were performed in polybrene-supplemented media (8 µg/ml). Media was replaced the following day and selection with 2 µg/mL puromycin was initiated two days post transduction.

PiggyBac transfection
The Super PiggyBac Transposase Expression Vector (System Biosciences, PB210PA-1) and CHARM-expressing *PiggyBac* transposon vector were co-transfected at a 1:10 molar ratio into N2a cells using TransIT-LT1 Transfection Reagent (Mirus Bio, 10767-122). Selection with 2 µg/mL puromycin was initiated 2 days post transfection. Cells were assessed for ZFcharm Kv1 and PrP expression using immunofluorescence staining (see below) followed by flow cytometry using the Attune NxT Flow Cytometer.

Immunofluorescence staining
Staining for cell surface proteins PrP, CD51, CD81, and CD151 was performed on cells at 50-90% confluency in 24-well plates. Cells were resuspended in PBS using mechanical force and transferred to a 96-well V-bottom plate. Cells were incubated at 4°C in the dark for 30 minutes with the appropriate fluorophore-conjugated antibody (Alexa Fluor 647 6D11 anti-PrP, also called anti-CD230, Biolegend, 808007; APC anti-human CD81, Biolegend, 349509; APC anti-human CD55, Biolegend, 311311; APC anti-human CD151, Biolegend, 350405) at a concentration of 0.5ug/mL. Cells were washed twice in PBS supplemented with 5% FBS and read out on the Attune NxT Flow Cytometer.

Cell viability staining
To assess cytotoxicity of the different epi-editors, HEK293T cells were transiently transfected with ZFP expressing constructs followed by FACS on TagBFP expression two days later. After recovering from FACS for four days, 1e6 cells were trypsinized, spun down at 400xg for 5 minutes, and resuspended in 1 mL of PBS. One µL of LIVE/DEAD™ Fixable Near-IR Dead Cell Stain for 633 or 635 nm excitation (Invitrogen™ L34975) dissolved in DMSO was added to the cells and kept on ice for 30 minutes protected from light. Cells were pelleted and washed with PBS twice followed by resuspension in 150 µL of PBS and flow cytometry on the Attune NxT Flow Cytometer. Total viable cells per 100 µL were counted based on near-IR (~780 nm) fluorescence.

Generation of genetic knockout cell lines
To knock out *DNMT3A* and *DNMT3B* in our mScarlet-CLTA reporter HEK293T cell line, we nucleofected Alt-R™ S.p. HiFi Cas9 Nuclease V3, 100 µg (IDT 1081060) complexed with guide RNA to form ribonucleoprotein (RNP). Guide RNAs designed using CRISPick software (*102*) were formed by mixing 5 µL Alt-R® CRISPR-Cas9 crRNA at 100 µM (IDT custom designs;

Table S1) and 5 µL Alt-R® CRISPR-Cas9 tracrRNA at 100 µM (IDT 1072533) and boiling at 95 C for 5 minutes followed by cooling to room temperature. To make RNP, 1.4 µL of the 50 µM annealed guide RNA was mixed with 1 µL of the Cas9 nuclease (62 µM) and 0.6 µL of phosphate buffered saline to a final volume of 3 µL, which was incubated at room temperature for 20 minutes and then placed on ice until ready for electroporation. HEK293T reporter cells were dissociated with trypsin and 1e6 cells were spun down. These were resuspended in Amaxa™ SF cell line 4D-nucleofector buffer with Cas9 RNP added and nucleofected in a 100 µL cuvette using program CM-130 following manufacturer's protocols (V4XC-2024, Lonza). Cells were immediately plated in a 6-well dish containing pre-warmed media (see culture conditions above). Knockout efficiency was determined by seeding cells in a 96-well plate and lysing with 200 µL QuickExtract DNA Extraction Solution (SS000035-D2, Biosearch Technologies) following manufacturer protocols. Two µL of cell lysate was used as a template for a 40 µL PCR using 2x Super Pfx Mastermix (CW2965, Cowin Biosciences) and the following primers: CAGCCAGGCTCCTAGACCCA (*DNMT3A*, Fwd), GGCGGGGTCATGTCTTCAGG (*DNMT3A*, Rev), TGGCAGGAAAAACCCCGTGT (*DNMT3B*, Fwd), and AGCCGTTCCCTATACATGAGTTCT (*DNMT3B*, Rev) (5' to 3') to generate a 715 bp amplicon for *DNMT3A* and a 700 bp amplicon for *DNMT3B*. PCRs were purified using QIAquick PCR Purification Kit (28104, Qiagen) and Sanger sequenced by Quintara Bio. Insertion/deletion (indel) frequencies were determined from Sanger traces using Synthego ICE analysis (https://ice.synthego.com/#/).

DNMT3L phylogeny construction
Genome-mining for DNMT3L orthologs and ancestral reconstructions was performed based on previously established methods (*61*). A list of ~200 *DNMT3L* orthologs was obtained by performing a BLASTP (*103*) search in the NCBI non-redundant protein sequences database, using the human and mouse DNMT3L amino acid sequences as a query, and removing sequences with >97% pairwise identity. A MAFFT multiple sequence alignment was performed using the FFT-NS-i (standard) strategy with a maximum of two iterations (*104*) and then used for phylogenetic tree construction implementing IQ-TREE software (*105*). With IQ-TREE we inferred the phylogenetic tree using the predicted best-fit model and ultrafast bootstrapping with 1000 replicates and optimized parameters. After visualization of the tree using the interactive tree of life (iTOL) v5 online tool (*106*), selected ancestral nodes were predicted with the IQ-TREE ASR function (*105*). Two dozen GenScript codon-optimized orthologs and ASRs were synthesized as DNA eBlocks (IDT). D3L sequences can be found in Table S2.

TALE Design
TALE DNA-binding domains were constructed following published guidelines (*107, 108*). In brief, potential 18-nucleotide binding sites beginning with the invariable thymine were compiled from the mouse and human *PRNP* promoter regions and scored for specificity using nucleotide BLAST (*103*). Top candidates were selected for synthesis in the chimerized TALE scaffold (*63*) using the following repeat variable diresidues (RVDs): HD for cytosine, NG for thymine, NI for adenine, NH for guanine, and G* for any possible 5-methyl-cytosine within a CpG dinucleotide. Each TALE was synthesized as eBlocks (IDT) in two halves which were cloned into a CHARM acceptor vector using NEBuilder HiFi DNA Assembly (New England BioLabs, E2621L). TALE sequences can be found in Table S2.

Extraction of HMW gDNA
To extract high molecular weight (HMW) genomic DNA (gDNA) from cells for Nanopore long-read sequencing analysis of CpG methylation, $1e^6$ cells were pelleted at 400xg for 5 minutes, rinsed with PBS, and pelleted again. Pellets were processed using the Monarch® HMW DNA Extraction Kit for Cells & Blood (New England Biolabs, T3050L). To extract HMW gDNA from mouse brain tissue, two 150 µm coronal sections were cut from flash-frozen hemispheres embedded in optimal cutting temperature (O.C.T.) compound (see below) and collected in a single 1.5 mL Eppendorf tube. These were frozen at -80°C until ready for preparation. Prior to processing using the Monarch® HMW DNA Extraction Kit for Tissue (New England Biolabs, T3060L), these sections were rinsed with ice-cold PBS twice and pelleted on a tabletop microcentrifuge (MyFuge 12 Mini Centrifuge, Benchmark Scientific C1012) to remove excess O.C.T. The gDNA extraction was performed following manufacturer instructions with slight modifications to maximize yield; three glass beads were used instead of two, and gDNA was eluted in 200 µL of water heated to 65°C. To concentrate the gDNA for Nanopore library preparation (to ~5 µg DNA in <24 µL), gDNA in the eluate was precipitated by adding 2 µL 20 mg/mL glycogen (Thermo Scientific, R0561), 22 µL 3M pH 5.2 sodium acetate, and 155 µL pure room temperature isopropanol followed by mixing and centrifugation at 15,000xg for 20 minutes at 4°C. Supernatant was carefully decanted and DNA pellets were washed with 1 mL 70% ethanol and centrifuged at 15,000xg again for 10 minutes at 4°C. Supernatant was decanted and the pellet was air-dried for 10 minutes. The DNA pellet was redissolved in 25 µL water at 56°C for two hours. Wide-bore pipette tips (Genesee Scientific, 22-427 and 22-424) were used for all gDNA handling steps to prevent shearing.

Target enrichment and Nanopore library preparation
Two upstream and two downstream guide RNAs were designed flanking the *PRNP* locus in a ~5 kb window using CHOPCHOPv3 (*109*). Alt-R® CRISPR-Cas9 tracrRNA (IDT, 1072533) and custom Alt-R® CRISPR-Cas9 crRNA (IDT) were annealed at 10 µM in nuclease-free duplex buffer (IDT, 11-01-03-01). In a 1.5 mL Eppendorf tube, 79.2 µL of water was combined with 10 µL of reaction buffer (RB) from the Cas9 Sequence Kit Cas9 Sequencing Kit (Oxford Nanopore Technologies, SQK-CS9109), 10 µL of 10 µM pooled annealed guide RNAs, and 0.8 µL of 62 µM Cas9 nuclease (Alt-R™ S.p. HiFi Cas9 Nuclease V3, IDT 1081060) and was complexed at room temperature for 30 minutes before use. Prior to Nanopore sequencing of native DNA molecules, the prion locus was enriched using 5 µg of input gDNA and prepared for sequencing following manufacturer's protocols (ONT, SQK-CS9109).

Nanopore sequencing
Target-enriched Nanopore libraries were loaded into a MinION Flow Cell R9.4.1 (ONT, FLO-MIN106D) after priming with the flow cell priming kit (ONT, EXP-FLP002) and sequenced on a MinION sequencing device (ONT, MIN-101B) with MinKNOW software (ONT, v.23.04.6) using fast base calling combined with adaptive sampling to further enrich the target locus. Live base calling was enabled by hardware (13th Gen Intel Core i7-13700 2.10 GHz and 16 GB RAM) with a GPU (NVIDIA, GeForce RTX 3070) running Windows 11 Pro. Each library was sequenced in series, and the flow cell was washed using the flow cell wash kit (ONT, EXP-WSH004) between samples. Each flow cell could run 3-4 libraries before requiring replacement. Two biological replicates were sequenced for each sample type.

Nanopore base calling and data analysis
Base calling was performed on the raw FAST5 files with Guppy (ONT, v.6.5.7), using a configuration file for high-accuracy modified DNA base calling on an R9.4.1 pore at 450 bases s^{-1}. The resulting reads were then mapped to the GRCh38 (human) or GRCm39 (mouse) reference genome without alternate contigs using minimap2 v.2.26 with default settings for alignment of nanopore reads (-x map-ont). Reads were filtered based on reciprocal 90% coverage with the target locus using the bedtools v.2.31.0 intersect (-wo -f 0.9 -r) command. Filtered, sorted, and indexed bam output files were used for methylation visualization (see below) or further processed using modkit tools (ONT, https://github.com/nanoporetech/modkit) and custom python scripts implementing Numpy v.1.26.3, Pandas v.2.2.0, and Seaborn v.0.13.2 for Pearson correlation and average methylation plots. Virtual environment files and custom scripts can be found here: https://github.com/edwin-n-neumann/CHARM_x_Prion.

Visualization of DNA methylation on individual Nanopore reads
Output bam files with read names numbered by average methylation were indexed and loaded into Integrative Genomics Viewer (*110*) v2.16.2 with the following settings: squished, small indel threshold <100 bp, hide mismatched bases, hide insertion markers, quick consensus mode, color by 5mC, sort by Read Name, Reverse Sort. To change colors to black (unmethylated) and blue (methylated), exported PNG files were adjusted with an Adobe Photoshop 2024 batch processing script for consistency.

RNA-seq analysis
N2a cells were maintained for 28 days post lentiviral transduction of ZFcharm Kv1 or CRISPRcharm Kv1 constructs. CRISPRcharm Kv1 was introduced into cells already expressing either a non-targeting sgRNA or a sgRNA targeting *Prnp.* An empty lentiviral vector was used as a no-editor control. Each transduction was done in triplicate. Cells were dislodged from 6-well plates using Trizol and total RNA was extracted using the Direct-zol RNA Miniprep Kit (Zymo, R2051). Libraries were prepared using the KAPA RNA HyperPrep Kit with RiboErase (HMR) (Roche, KK8560) and sequenced as 50 bp single-end reads on a NovaSeq 6000 (Illumina). Raw sequencing reads were aligned to the mouse genome (mm39) using STAR 2.7.1a and quantified using featureCounts 1.6.2 (*111*). Differential expression analysis was carried out using DESeq2 (*112*) using default parameters. The lfcShrink function was applied using the apeglm shrinkage estimator. Sequencing data are available on GEO (GSE255987).

Clonal bisulfite sequencing
Clonal bisulfite sequencing (*32*) of the EFS promoter was performed on (1) genomic DNA extracted from lentivirally transduced N2a cells, (2) double-stranded AAV genomes extracted from brain homogenate, or (3) single-stranded AAV genomes extracted from viral particles. N2a genomic DNA was extracted using the PureLink Genomic DNA Mini Kit (Invitrogen, K182001). AAV episomal DNA was obtained via Trizol-Chloroform extraction from brain homogenate followed by treatment with T5 exonuclease (New England BioLabs, M0663S) and RNase Cocktail Enzyme Mix (Thermo Fisher Scientific, AM2288). To extract single-stranded AAV DNA, viral particles were treated with Turbonuclease (MilliporeSigma, T4330) to digest contaminating plasmid DNA and then with Proteinase K to digest viral capsids. Both double- and single-stranded AAV DNA was purified with the DNA Clean & Concentrator-5 Kit (Zymo, 11-302B). Bisulfite conversion was performed on 100-500 ng DNA using the EZ DNA

Methylation Lightning Kit (Zymo, D5001). Purified bisulfite-converted DNA was amplified with forward primer GAGTGGTTAATTTTATTATTAGGGGT (5′ to 3′) and reverse primer TTTCTAACAATTTATTTAATCCTAACCA (5′ to 3′) using EpiMark Hot Start Taq (New England BioLabs, M0490S), and purified using a QIAquick PCR Purification Kit (QIAGEN, 28104). Amplicons were cloned into pCR2.1-TOPO Vector using a TOPO TA Cloning Kit (Invitrogen, 451641) and transformed into Stellar Competent E. coli Cells (Takara Bio, 636766). Cells were plated on plates supplemented with carbenicillin, X-gal, and IPTG for blue-white screening. Colonies were sequenced by Sanger sequencing and reads were processed for display using QUMA software (*114*).

AAV production and titering

Recombinant AAVs (AAV-PHP.eB) were produced in suspension HEK293T cells, using F17 media (Thermofisher, A138501). Cell suspensions were incubated at 37°C, 8% CO_2, 80 RPM. 24 hours before transfection, cells were seeded in 500–1000 mL at ~1 million cells/mL. The day after, cells (~2 million cells/mL) were transfected with pHelper, pRepCap, and pTransgene (2:1:1 ratio, 2 µg total DNA per million cells) using Transport 5 transfection reagent (Polysciences, 26008-50) with a 2:1 PEI:DNA ratio. Three days post-transfection, cells were pelleted at 2000 RPM for 12 minutes into Nalgene conical bottles. The supernatant was discarded, and cell pellets were stored at -20°C until purification. Each pellet, corresponding to 500 mL of cell culture, was resuspended in 14 mL of 500 mM NaCl, 40 mM Tris-base, 10 mM $MgCl_2$, with Salt Active Nuclease (ArcticZymes, #70920-202) at 100 U/mL. Afterwards, the lysate was clarified at 5000 RCF for 20 minutes and loaded onto a density step gradient containing OptiPrep (Cosmo Bio, AXS-1114542) at 60%, 40%, 25%, and 15% at a volume of 6, 6, 8, and 5 mL, respectively, in OptiSeal tubes (Beckman, 342414). The step gradients were spun in a Beckman Type 70ti rotor (Beckman, 337922) in a Sorvall WX+ ultracentrifuge (Thermo Scientific, 75000090) at 67,000 RPM for 75 minutes at 18°C. Afterwards, ~4.5 mL of the 40–60% interface was extracted using a 16-gauge needle, filtered through a 0.22 µm PES filter, buffer exchanged with 100K MWCO protein concentrators (Thermo Scientific, 88532) into PBS containing 0.001% Pluronic F-68, and concentrated down to a volume of 200–1000 µL. The concentrated virus was filtered through a 0.22 µm PES filter and stored at 4°C or -80°C.

To determine AAV titers, 5 µL of each purified virus library was incubated with 100 µL of an endonuclease cocktail consisting of 1000U/mL Turbonuclease (Sigma T4330-50KU) with 1X DNase I reaction buffer (New England BioLabs, B0303S) in UltraPure DNase/RNase-Free distilled water at 37°C for one hour. Next, the endonuclease solution was inactivated by adding 5 µL of 0.5 M EDTA, pH 8.0 (ThermoFisher Scientific, 15575020) and incubated at room temperature for 5 minutes and then at 70°C for 10 minutes. To release the encapsidated AAV genomes, 120 µL of a Proteinase K cocktail consisting of 1 M NaCl, 1% N-lauroylsarcosine, 100 µg/mL Proteinase K (QIAGEN, 19131) in UltraPure DNase/RNase-Free distilled water was added to the mixture and incubated at 56°C for 2–16 hours. The Proteinase K-treated samples were then heat-inactivated at 95°C for 10 minutes. The released AAV genomes were serial diluted between 460–4,600,000X in dilution buffer consisting of 10X PCR Buffer (Thermo Fisher Scientific, N8080129), 2 µg/mL sheared salmon sperm DNA (Thermo Fisher Scientific, AM9680), and 0.05% Pluronic F68 (Thermo Fisher Scientific, 24040032) in UltraPure Water (Thermo Fisher Scientific). 2 µL of the diluted samples were used as input in a ddPCR supermix (Bio-Rad, 1863023). Primers and probes, targeting the ITR region, were used for titration at a

final concentration of 900 nM and 250 nM (ITR2_Forward: 5'-GGAACCCCTAGTGATGGAGTT-3'; ITR2_Reverse: 5'-CGGCCTCAGTGAGCGA-3'). The droplets were transferred to the thermocycler and cycled according to the manufacturer's protocol with an annealing/extension of 58°C for one minute. Finally, droplets were read on a QX100 Droplet Digital System to determine titers.

Mice

All in vivo experiments were approved by the Institutional Animal Care and Use Committee of the Broad Institute (Protocol #0162-05-16-2, most recent approval date: 2024-01-03) and were performed in accordance with the National Institutes of Health *Guide for the Care and Use of Laboratory Animals*. Experiments in this study used 192 C57BL/6N mice (90 female, 102 male) obtained from Charles River Laboratories. Unless otherwise noted, mice were between 5-8 weeks old at the time of AAV injections.

Intravenous AAV injection

Mice were anesthetized using inhaled isoflurane at 1-3%. AAV vectors (0.75e13, 1.5e13, or 3e13 vg/kg, ~100ml injection volume) were administered intravenously into the right retro-orbital sinus of the animal using a 300 µL insulin syringe with a 31G needle (328438, Becton Dickinson, USA) following established protocols (*64, 113*). One drop of 0.5% proparacaine (07-892-9554, Patterson Veterinary, USA) was applied topically to the eye immediately following injection. Mice were euthanized using CO_2 inhalation at timepoints of 6- or 13-weeks post-injection, following which the brains were harvested and cut in half. One hemisphere was placed in a microtube and flash-frozen on dry ice for ELISA (see below), while the other hemisphere was prepared for histological analysis. In brief, a small amount of optimal cutting temperature (OCT) compound (Tissue-Tek 4583, Sakura, USA) was placed into a 15x15x5 mm cryomold (Tissue-Tek 4566, Sakura, USA), the hemisphere was placed cut side down into the mold, and fully covered with additional OCT compound prior to being flash-frozen on dry ice. All samples were stored at -80°C until further processing.

Mouse perfusions

Mice were deeply anesthetized under 2-5% isoflurane and 0.5-1 LPM oxygen in an induction chamber. Mice were then transferred to a nose cone providing 2-5% isoflurane and 0.5-1 LPM oxygen. Anesthesia depth was validated with lack of bilateral toe pinch prior to the start of the surgical procedure. Mice were continuously monitored throughout the procedure for any signs of responsiveness. Paw color and respiration rate were monitored at all times during anesthesia. Once anesthesia was stable and at an acceptable plane for surgery (based on lack of a toe-pinch and eye blink response, and stable slow respiratory rate), an incision was made through the skin below the ribcage and blunt dissection scissors were used to separate the outer layers of skin from the cavity wall. A mid-sternal thoracotomy was then performed to expose the heart and great vessels. Perfusate was delivered using a needle through the left ventricle and an incision was made in the right atrium to provide an outflow for blood and perfused fluids.
Perfusion was carried out with ice-cold saline solution followed by phosphate buffered saline containing 4% paraformaldehdye (PFA). Perfusion was complete when outflow perfusate showed no visual trace of blood, and the animal had no cardiac or respiratory activity. Mice were decapitated prior to brain dissection.

Brain homogenization

One hemisphere was homogenized at 10% wt/vol in cold 0.2% CHAPS solution prepared in 1X PBS with 1 tablet protease inhibitor (Roche cOmplete 4693159001, Millipore Sigma, USA) per 10 mL in 7 mL tubes pre-loaded with zirconium oxide beads (Precellys , Bertin, USA), using 3 x 40 second pulses on a Bertin MiniLysis Homogenizer (Bertin, USA). Homogenate was aliquoted into 40 µL aliquots for protein analysis and 300 µL aliquots for qPCR analysis, and stored at -80°C until further analysis.

Protein analysis

PrP concentration in the brain was quantified using a previously published PrP ELISA (*70*). Briefly, the assay uses EP1802Y antibody (ab52604, Abcam, USA) for capture and biotinylated 8H4 antibody (ab61409, Abcam, USA) for detection, with streptavidin-HRP (Pierce High Sensitivity, 21130, Thermo Fisher Scientific, USA) and TMB substrate (7004P4, Cell Signaling Technology, USA). Recombinant mouse PrP (MoPrP23-231) prepared as described (*115*) was used for a standard curve. Protein knockdown was calculated by dividing the concentration of residual PrP in each treatment brain, by the mean concentration of residual PrP in the saline control brains from the same time point.

RT-qPCR

Mouse *Prnp* RNA was quantified using RT-qPCR. RNA extracts were treated with DNase I (New England BioLabs, M0303S). Library preparation was performed using the RevertAid First Strand Synthesis Kit (Thermo Fisher Scientific, K1691). Taqman qPCR (Thermo Fisher Scientific, 4331182) was performed on cDNA samples using the QuantStudio 7 Flex (Applied Biosystems). ΔΔCt values were calculated based on the amplification of *Gapdh*, and normalized to the mean of the no injection controls. Probe and quencher sequences were purchased from Fisher Scientific as premixed Gene Expression Assays (*Gapdh* control, ID Mm99999915_g1; *Prnp* target, ID Mm07296968_m1).

Tissue processing and sectioning

Whole mouse brains harvested from perfused mice were incubated overnight at 4°C in 4% PFA. Fixed brains were then washed in 1X PBS and dehydrated overnight at 4°C in 30% sucrose, followed by a second overnight incubation at 4°C in a 1:1 mixture of 30% sucrose and O.C.T. compound (Tissue-Tek, 4583). Dehydrated brains were placed in cryomolds containing O.C.T. and snap-frozen in liquid nitrogen-chilled isopentane. 10 µm coronal brain sections were cut using a Leica CM3050 S Research Cryostat and placed on SuperFrost Plus slides (VWR, 48311-703). Brains used to extract DNA for Nanopore long-read sequencing were harvested from non-perfused mice and directly embedded in O.C.T. before freezing on dry ice. These were cut into 150 µm sections using a Leica CM3050 S Research Cryostat and stored in tubes at -80°C before use.

Hybridization chain reaction RNA fluorescence in situ hybridization (HCR RNA-FISH)

Coronal brain sections on SuperFrost Plus slides were immersed in 4% PFA at 4°C for 15 minutes and then sequentially immersed in 50% ethanol, 70% ethanol, 100% ethanol, and 1X PBS at room temperature for 5 minutes. A hydrophobic barrier was drawn around the tissue using an ImmEdge™ Hydrophobic Barrier Pen (Vector Laboratories, 101098-065). Third-generation multiplexed HCR RNA-FISH was performed as previously described (*116*).

Briefly, tissue samples were pre-hybridized in hybridization buffer (Molecular Instruments) at 37°C for 10 minutes and then incubated in a 37°C humidified chamber overnight with split-initiator probes hybridizing to the *Prnp* and *Uchl1* mRNA transcripts diluted to a concentration of 4 nM in Hybridization Buffer. Split-initiator probes were purchased from Molecular Technologies. The slides were then immersed in 75%, 50%, and 25% probe wash buffer (Molecular Instruments) solutions at 37°C for 15 minutes, followed by two incubations in 5X SSCT, one for 15 minutes at 37°C and another for 5 minutes at room temperature. Tissue sections were then equilibrated in amplification buffer (Molecular Instruments) for 30 minutes at room temperature. Separately, metastable fluorescent hairpins conjugated to Alexa Fluor 647 and Alexa Fluor 546 were snap-cooled and diluted to 60 nM in amplification buffer. Samples were incubated in hairpin solution overnight in a dark humidified chamber at room temperature. Excess hairpin amplifiers were removed the next day in 5X SSCT at room temperature before staining with 1 µg/mL DAPI for 10 minutes, washing again in 5X SSCT, and mounting in VECTASHIELD® PLUS Antifade Mounting Medium (Vector Laboratories, H-1900). Brain sections were imaged as z-stack tile scans on a Zeiss LSM 980 with Airyscan 2 Laser Scanning Confocal with a 20X objective.

Image analysis
Maximum orthogonal projections and stitching of z-stack tile scales was performed using ZEN Blue software (Zeiss). Cell detection and classification was carried out using QuPath software v0.5.0 (*71*). Briefly, cells were detected using QuPath's cell detection tool on the DAPI channel (cell expansion = 4 µm). QuPath's built-in machine learning classification tool was used to detect neurons (using *Uchl1*-Alexa Fluor 647 signal) and *Prnp*+ cells (using *Prnp*-Alexa Fluor 546 signal). Multiple images were used to train the classifiers. Zoomed-in images of brain regions were median filtered using Fiji software v2.9.0 (*117*).

Statistical analyses
All statistical tests performed in this study are indicated in the figure legends.

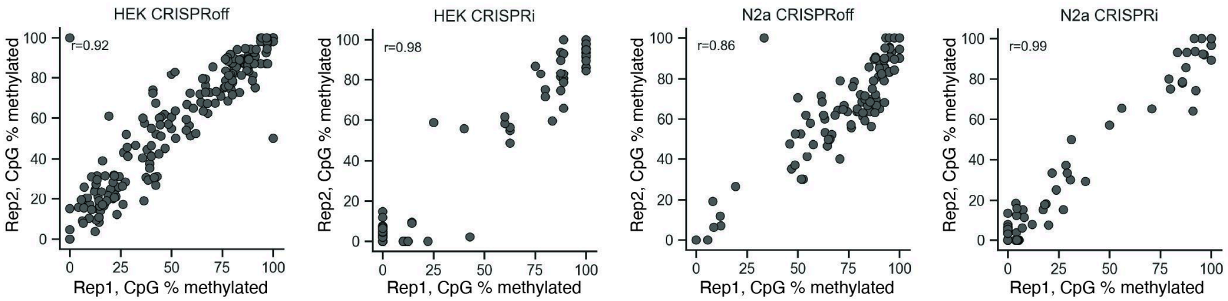

Fig. S1. Reproducibility of in vitro Nanopore analysis. Nanopore was performed in two replicates (corresponding to Figure 1C-D) and Pearson correlation coefficient is shown. These data indicate the percent of reads methylated at each CpG position is similar across replicates.

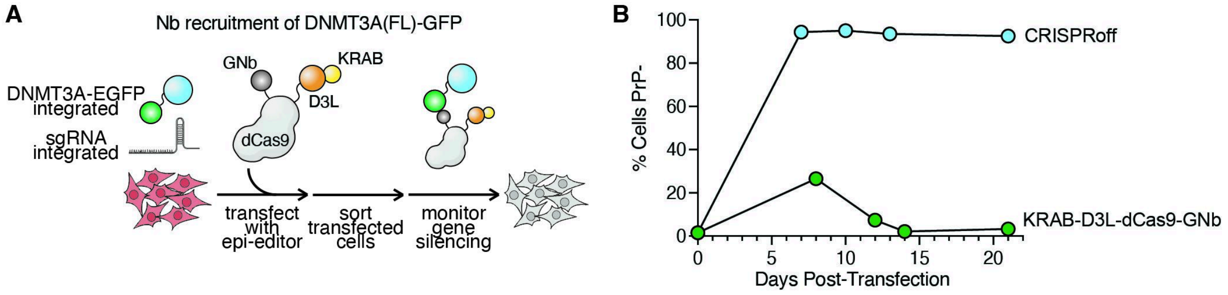

Fig. S2. Full length DNMT3A recruitment. (**A**) HEK293T cells containing the mScarlet-*CLTA* reporter and constitutively expressing sgRNA targeting the *CLTA* TSS and a full length DNMT3A-EGFP fusion were transfected with an effector fusion of dCas9, D3L, KRAB, and the GFP nanobody (GNb). (**B**) Cells were monitored for reporter silencing by flow cytometry. Data are mean ± SEM of n=2 replicates.

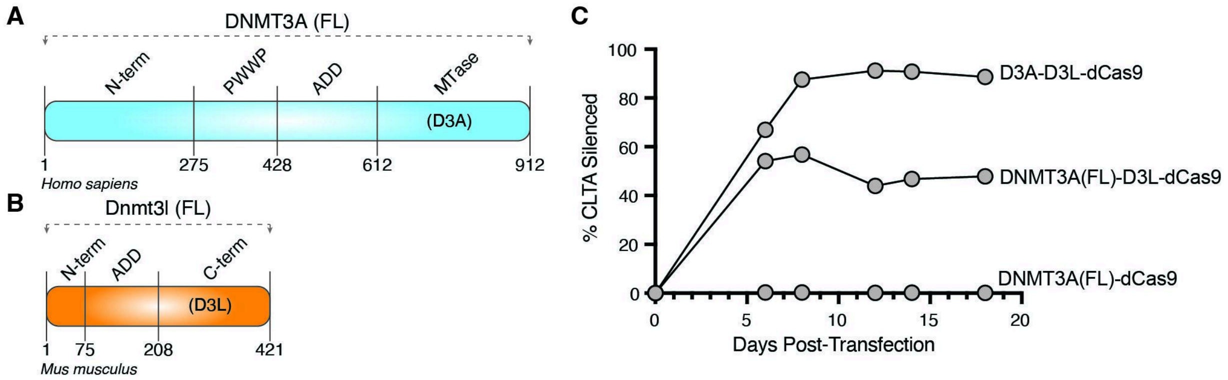

Fig. S3. Full length de novo methyltransferase domain regulation. (**A**) Schematic of DNMT3A1 domains. (**B**) Schematic of DNMT3L domains. (**C**) HEK293T cells containing the mScarlet-*CLTA* reporter and sgRNA against the *CLTA* TSS were targeted by effectors containing full length (FL) or catalytic-only (D3A) fusions of DNMT3A with D3L-dCas9 and monitored for silencing by flow cytometry. Data are mean ± SEM of n=2 replicates.

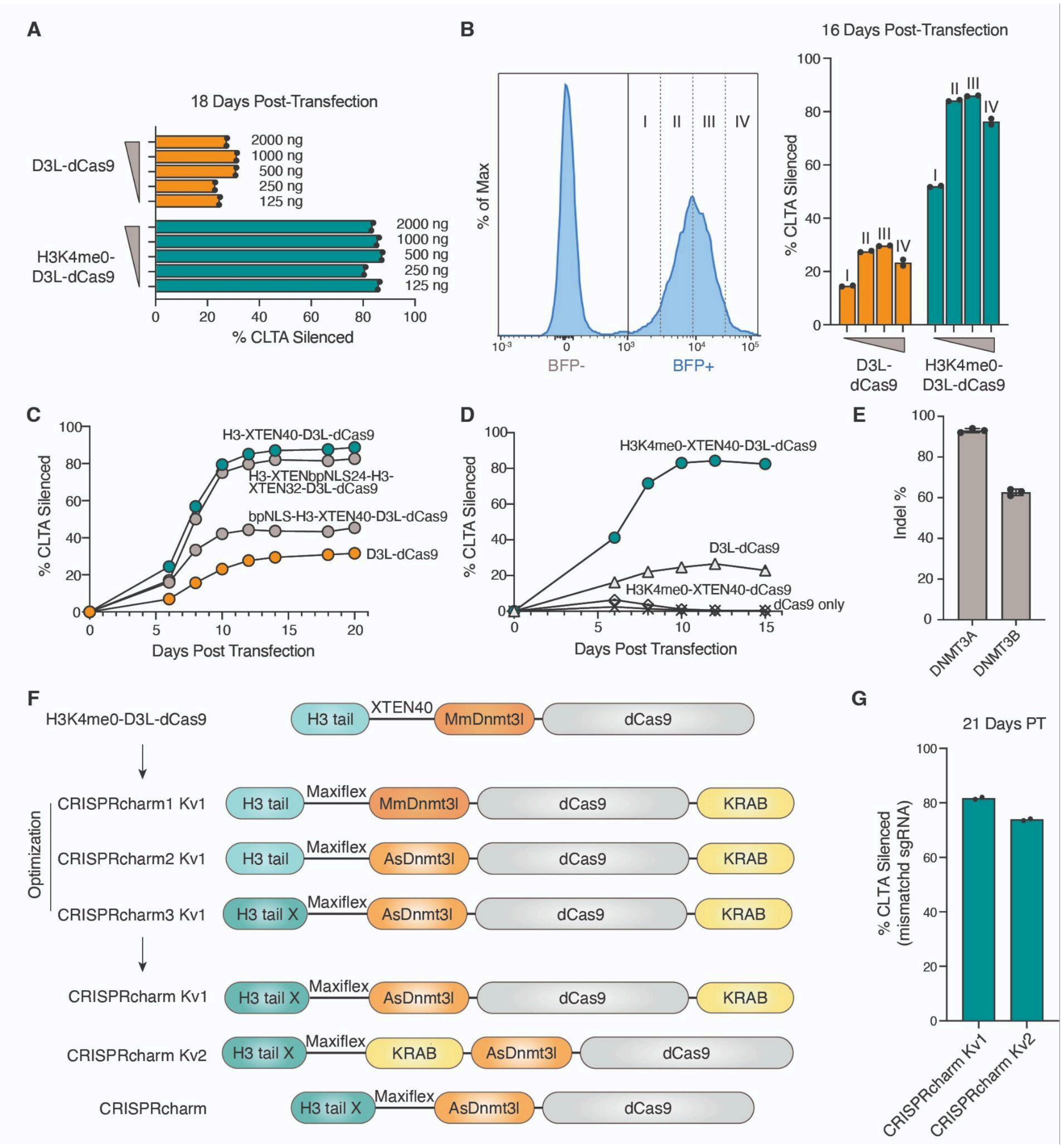

Fig. S4. CHARM dose-titration and optimization schematics. (**A**) Cells were transiently transfected with different nanogram (ng) amounts of plasmid DNA encoding D3L-dCas9 or CHARM effectors. (**B**) Cells were transiently transfected with plasmids encoding D3L-dCas9 or CHARM effectors and sorted into four bins based on transgene expression level. (**C**) Cells were transiently transfected with different plasmids encoding D3L-dCas9 or CHARM effectors with varying N-terminal appendages. Data are mean ± SEM of n=2 replicates. (**D**) Cells were transiently transfected with H3 tail and D3L fusions to dCas9 indicating their effects are

synergistic rather than additive. (**E**) Indel frequencies of DNMT3A and DNMT3B polyclonal knockout in HEK293T cells with mScarlet-*CLTA* reporter. (**F**) Schematics of the full optimization history and nomenclature for CHARM effectors. H3 tail X refers to the 30 amino acid H3 tail. (**G**) Cells with a mismatched sgRNA against the *CLTA* TSS to improve dynamic range of silencing were transiently transfected with plasmids encoding either CRISPRcharm Kv1 or CRISPRcharm Kv2 effectors. All experiments shown are targeting the mScarlet-*CLTA* reporter in HEK293T cells.

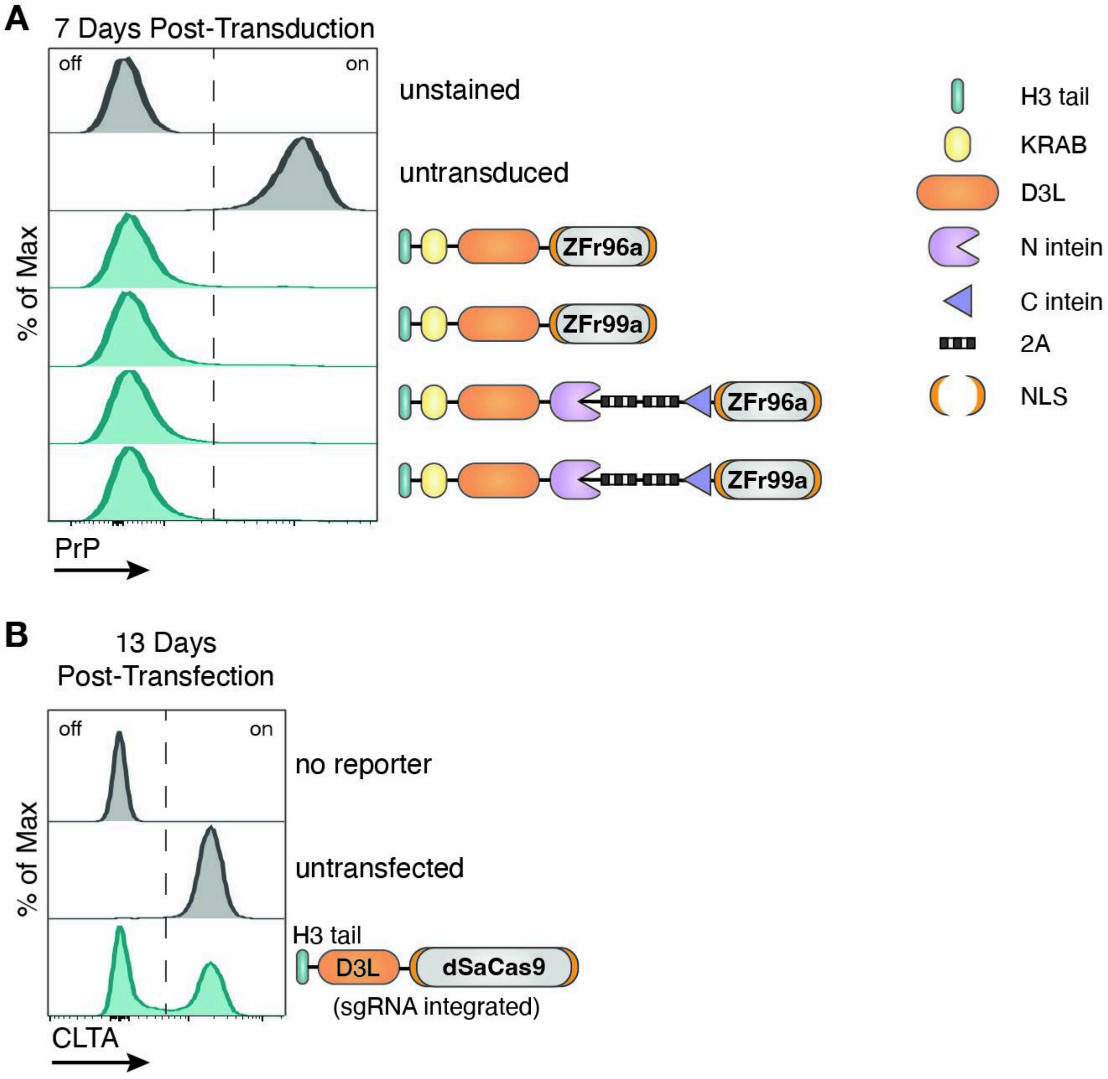

Fig. S5. CHARM variations using split-inteins or a small Cas effector. (**A**) HEK293T cells were transduced with lentivirus encoding ZFcharm Kv1 with ZFPs targeting the human *PRNP* TSS. Direct fusions and split-intein CHARMs are equally effective in PRNP repression as measured by Alexa Fluor 647 anti-PrP. (**B**) HEK293T cells containing the mScarlet-*CLTA* reporter and SaCas9 sgRNA against the *CLTA* TSS were transiently transfected with a CRISPRcharm construct using dSaCas9 as the DNA-binding domain. These were monitored for *CLTA* silencing by flow cytometry.

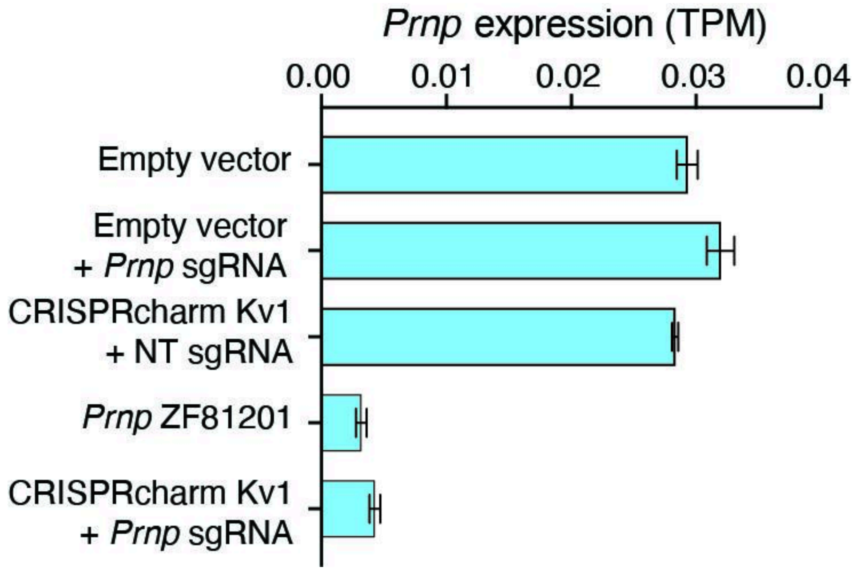

Fig. S6. Target repression quantified by RNAseq. Quantified *Prnp* RNA levels in N2a cells transduced with ZFcharm Kv1 or CRISPRcharm Kv1 lentiviral constructs corresponding to Fig. 3E and F). Data are mean ± SEM of n=3 replicates.

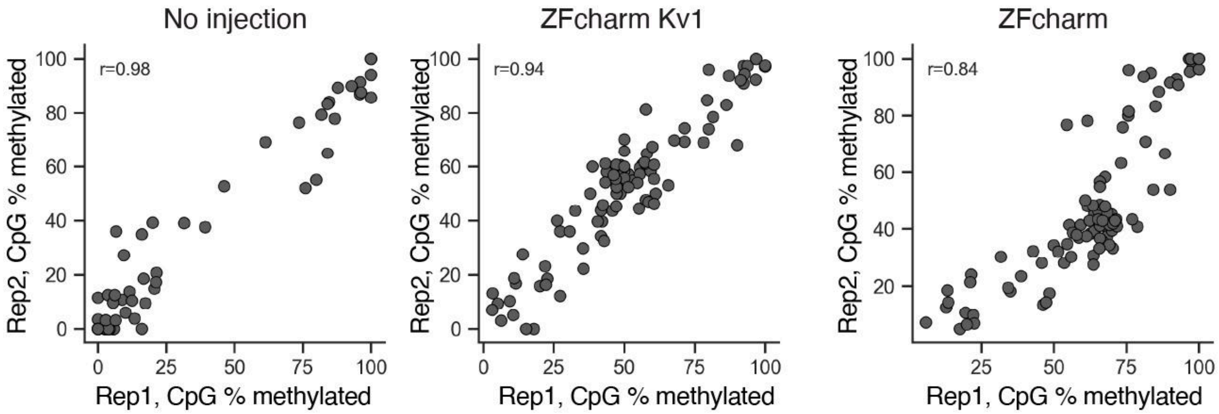

Fig. S7. Reproducibility of in vivo 5mCpG quantification using nanopore sequencing. % 5mCpG is compared between two biological replicates for each condition corresponding to Figure 4D. Pearson correlations are shown indicating the percent of reads methylated at each CpG position is similar across biological replicates.

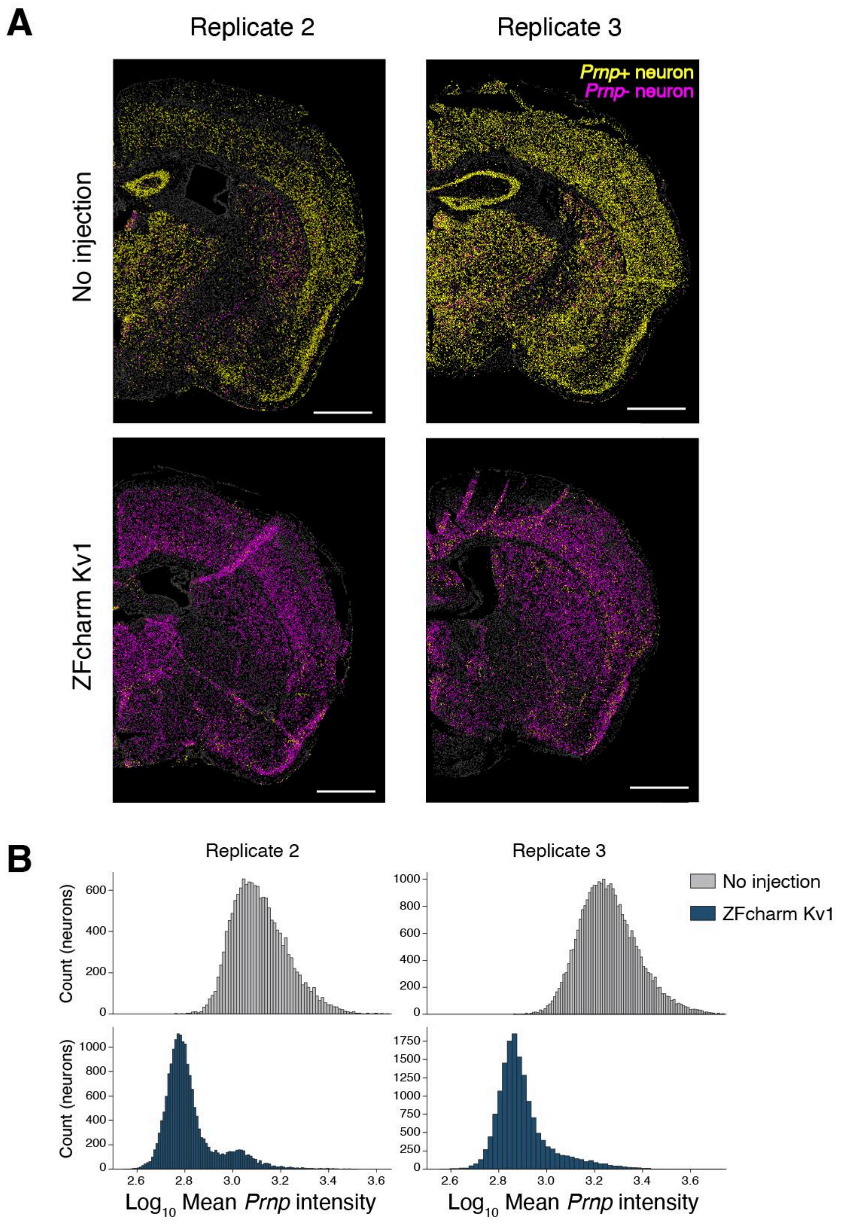

Fig. S8. AAV-delivered ZFcharm Kv1 silences the majority of neurons in the mouse brain. **(A)** Single-cell identification of *Prnp*+ (yellow) and *Prnp*- (magenta) neurons in additional 10 µm coronal brain hemisphere sections. *Prnp*-expressing *Uchl1*+ neurons were identified via machine learning classification using QuPath software (*71*). *Uchl1*- cells are shown in gray. Cell boundaries represent 4 µm expansions from DAPI-detected nuclei. Scale bar, 1mm. **(B)** Representative histograms of mean *Prnp* intensity in neurons.

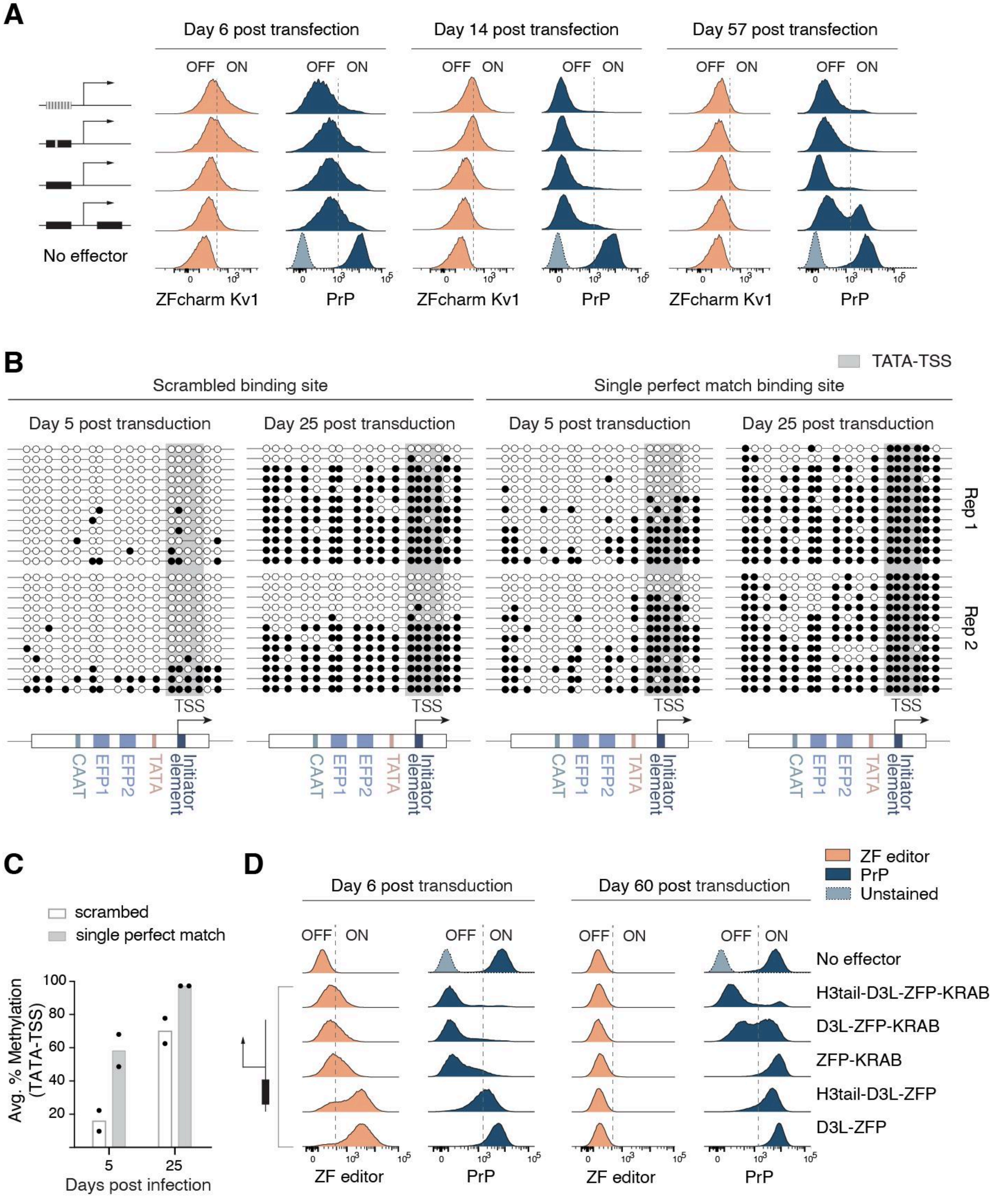

Fig. S9. Confirmation of CHARM self-silencing efficacy. (A) Representative ZFcharm Kv1 (orange) and PrP (navy) expression data over time following transfection of *piggyBac* donor and transposase plasmids in N2a cells. Dashed line indicates separation between expressing ('ON') and silenced ('OFF') cells. **(B)** Clonal bisulfite sequencing of EFS promoter driving ZFcharm

Kv1-SCR and ZF-charm Kv1-SPM expression 5 and 25 days post transduction of N2a cells. Each line is an individual PCR clone. Circles depict methylated (black) and unmethylated (white) CpG sites. Sequence elements within the EFS promoter are shown in the schematics under the data. CpGs between the TATA box and TSS are highlighted in gray. **(C)** Bar chart showing average % 5mCpG between the TATA box and TSS. **(D)** Representative flow cytometry histograms of ZF editor and PrP expression 6 and 60 days post infection across ZF-SPM constructs. Dashed line indicates separation between expressing ('ON') and silenced ('OFF') cells. Data are mean ± SD of n=2 replicates.

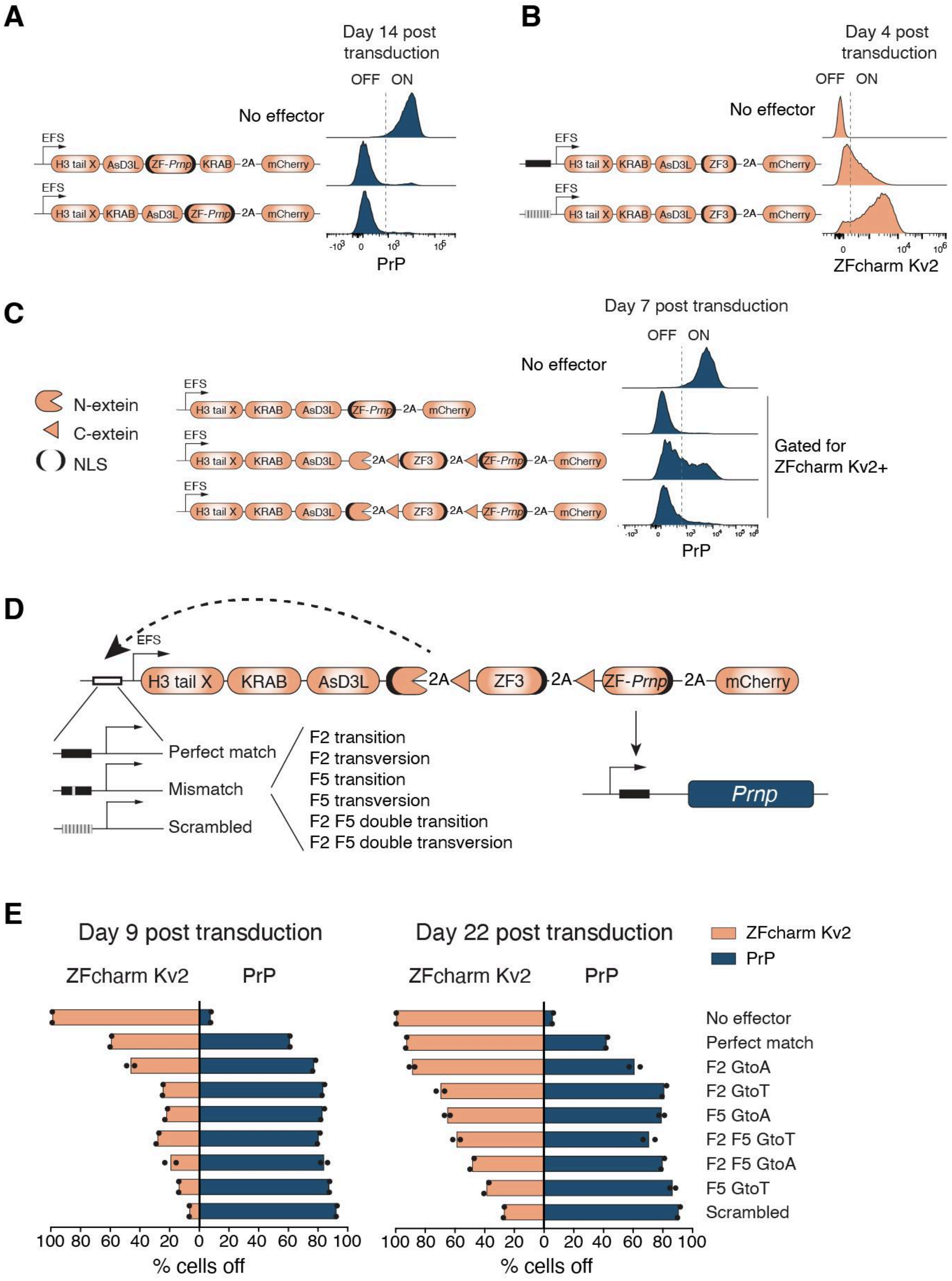

Fig. S10. Optimization of modular self-silencing CHARM construct. (A-C) Representative flow cytometry data of two replicates collected at the indicated time points after lentiviral transduction of N2a cells. Dashed line indicates separation between expressing ('ON') and silenced ('OFF') cells. **(A)** Placing the KRAB domain in the linker region between the H3 tail and D3L (ZFcharm Kv2) is tolerated when using ZF-mediated DNA binding, as shown above for dCas9-based targeting (fig. S5A). **(B)** ZFcharm Kv2 with a ZF3 DNA binding domain can

rapidly silence itself. **(C)** Placing NLS sequences on both the N- and C-exteins improves *Prnp* silencing. **(D)** Schematic of an alternative approach to tuning a modular self-silencing ZFcharm Kv2. **(E)** Introducing point mutations into the ZF3 binding site upstream of the EFS promoter slows the rate of self-silencing. Data are mean ± SD of n=2 replicates.

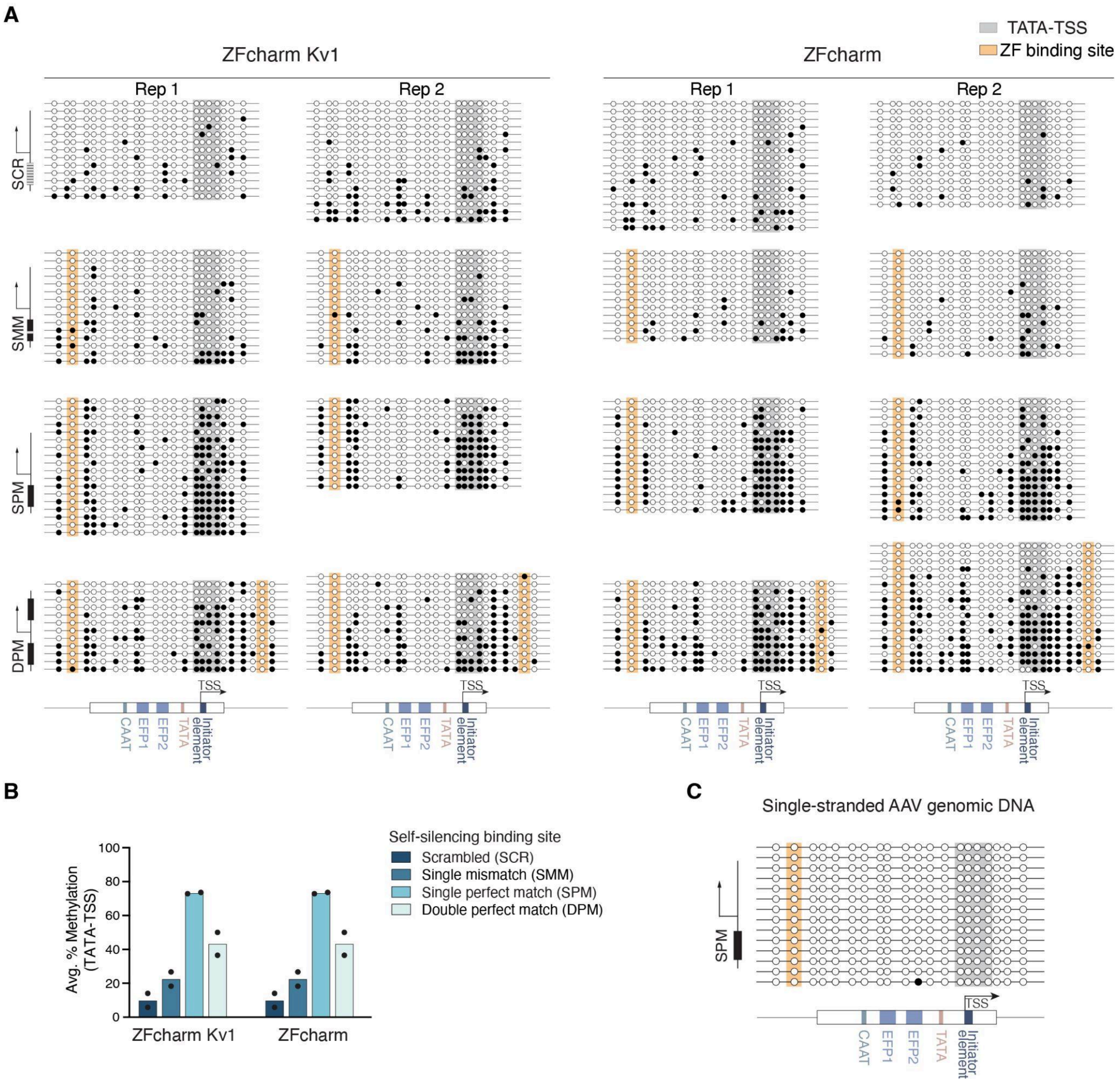

Fig. S11. The EFS promoter driving self-silencing CHARM becomes methylated in vivo. **(A)** Clonal bisulfite sequencing of EFS promoter driving self-silencing ZFcharm Kv1 and ZFcharm constructs in vivo. Each line is an individual PCR clone. Circles depict methylated (black) and unmethylated (white) CpG sites. Sequence elements within the EFS promoter are shown in the schematics under the data. CpGs between the TATA box and TSS are highlighted in gray and the ZF binding site is highlighted in orange. **(B)** Bar chart showing average % 5mCpG between the TATA box and TSS. Data are mean ± SD of n=2 replicates. **(C)** Clonal bisulfite sequencing of EFS promoter in single-stranded AAV genomic DNA extracted from ZFcharm Kv1-SPM virus.

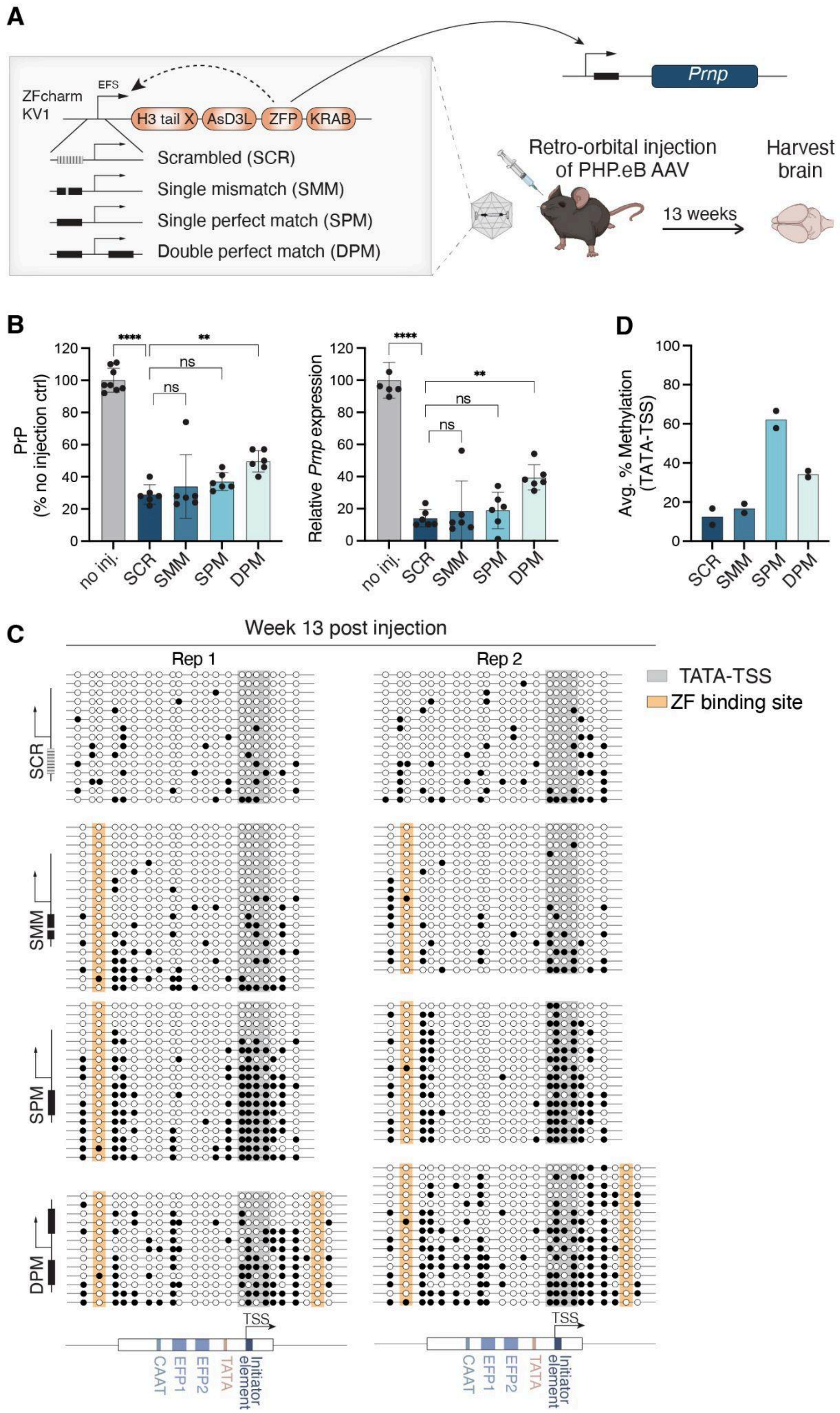
A
ZFcharm KV1
EFS
H3 tail X
AsD3L
ZFP
KRAB
Scrambled (SCR)
Single mismatch (SMM)
Single perfect match (SPM)
Double perfect match (DPM)
Prnp
Retro-orbital injection of PHP.eB AAV
Harvest brain
13 weeks
B
PrP (% no injection ctrl)
Relative Prnp expression
no inj.
SCR
SMM
SPM
DPM
ns
D
Avg. % Methylation (TATA-TSS)
C
Week 13 post injection
Rep 1
Rep 2
TATA-TSS
ZF binding site
TSS
CAAT
EFP1
EFP2
TATA
Initiator element

Fig. S12. *Prnp* silencing is stable following CHARM self-silencing in vivo. (A) Schematic of experimental design. **(B)** PrP ELISA and *Prnp* RT-qPCR data generated from whole brain hemisphere homogenate 13 weeks post injection of 1.5e13 vg/kg AAV. **(C)** Clonal bisulfite sequencing of the EFS promoter driving expression of self-silencing ZFcharm Kv1 13 weeks post AAV injection. Each line is an individual PCR clone. Circles depict methylated (black) and unmethylated (white) CpG sites. Sequence elements within the EFS promoter are shown in the schematic under the data. CpGs between the TATA box and TSS are highlighted in gray and the ZF binding site is highlighted in orange. Data are mean ± SD of n=5-8 replicates. Statistical analyses are one-way ANOVAs followed by Tukey's multiple comparisons test (** $p < 0.005$; **** $p<0.0001$; ns, not significant). **(D)** Bar chart showing average % 5mCpG between the TATA box and TSS. Data are mean ± SD of n=5-8 replicates.

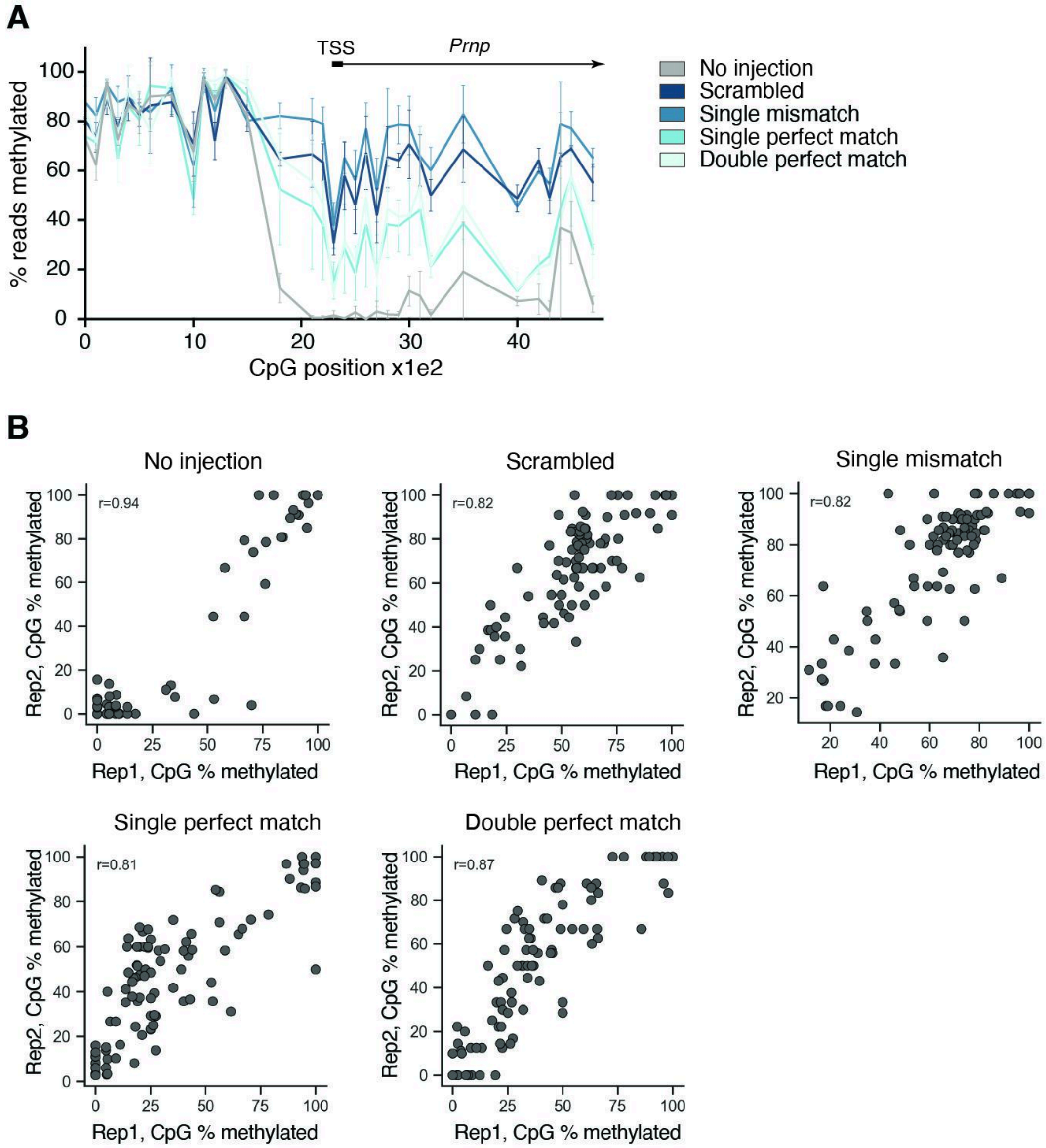

Fig. S13. Self-silencing ZFcharm Kv1 constructs methylate the *Prnp* promoter in vivo. (A) Target-enriched nanopore sequencing of *Prnp* promoter in brains harvested 13 weeks post AAV injection. Data are mean ± SD of n=2 replicates. **(B)** 5mCpG quantification via nanopore sequencing is reproducible. % 5mCpG is compared between two biological replicates for each condition. Pearson correlations are shown on each graph.

Table S1. CRISPR-Cas9 sgRNA sequences used in this study.

sgRNA Target	Spacer Sequence
CLTA TSS	TCCCAGTCGGCACCACA
CLTA TSS mismatched	CTCCGAGTCGGCACCACAG
CD55 TSS	CTGCGACTCGGCGGAGTCC
CD81 TSS	GAGAGCGAGCGCGCAACGG
CD151 TSS	GACAATGAGCAGGGTGTCC
Hs*PRNP* TSS	CCGAGGCAGGTAAACGCCCG
Mm*Prnp* TSS 1	GTCTGCTGATCCGACAACG
Mm*Prnp* TSS 2	CATTTAAGCCAGTCCGGAG
Mm*Prnp* TSS 3	TAGTTGCTGAGCGTCGTCA
Hs*PRNP* locus enrichment upstream 1	CACCCTTGGAGTGGTTCATA
Hs*PRNP* locus enrichment upstream 2	GTTACGTCAACAGCATACAG
Hs*PRNP* locus enrichment downstream 1	GCGTTCACGTTAGAGTAAGC
Hs*PRNP* locus enrichment downstream 2	GTCTGAGCTTTCCGTCTTCC
Mm*Prnp* locus enrichment upstream 1	GGCCTCCTTCCCTTATGAAT
Mm*Prnp* locus enrichment upstream 2	ACTTCGTAATAACACCCCAC
Mm*Prnp* locus enrichment downstream 1	TAGTGGTACCAGTCCAATTT
Mm*Prnp* locus enrichment downstream 2	GCGAAGTCAGCTTAATTCTA
CLTA TSS (SaCas9)	CGCTGTGGTGCCGACTGGGAG
DNMT3A knockout	GCGTACCAGTACGACGACGA
DNMT3B knockout	ACAGCTCAAGGAAGCGATCC
