# Supplementary material for: Brain-wide silencing of prion protein by AAV-mediated delivery of an engineered compact epigenetic editor
Source: Science. Author manuscript; Available in PMC 2025 Mar 3. (PMC11875203; doi:10.1126/science.ado7082)
Supplement: MDAR Checklist [file NIHMS2046549-supplement-MDAR_Checklist.pdf]

Materials Design Analysis Reporting (MDAR) Checklist for Authors

The MDAR framework establishes a minimum set of requirements in transparent reporting applicable to studies in the life sciences (see Statement of Task: doi:10.31222/osf.io/9sm4x.). The MDAR checklist is a tool for authors, editors and others seeking to adopt the MDAR framework for transparent reporting in manuscripts and other outputs.   Please refer to the MDAR Elaboration Document for additional context for the MDAR framework.

Materials

**Antibodies**	**Yes (indicate where provided: page no/section/legend)**	**n/a**
For commercial reagents, provide supplier name, catalogue number and RRID, if available.	Materials and Methods	

**Cell materials**	**Yes (indicate where provided: page no/section/legend)**	**n/a**
**Cell lines:** Provide species information, strain. Provide accession number in repository **OR** supplier name, catalog number, clone number, **OR** RRID	Materials and Methods: *"HEK293T (ATCC, CRL-3216) and Neuro-2a (N2a; ATCC, CCL-131) cells"*	
**Primary cultures:** Provide species, strain, sex of origin, genetic modification status.		x

**Experimental animals**	**Yes (indicate where provided: page no/section/legend)**	**n/a**
**Laboratory animals:** Provide species, strain, sex, age, genetic modification status. Provide accession number in repository **OR** supplier name, catalog number, clone number, **OR** RRID	Materials and Methods: *"Experiments in this study used 192 C57BL/6N mice (90 female, 102 male) obtained from Charles River Laboratories"*	
**Animal observed in or captured from the field:** Provide species, sex and age where possible		X
**Model organisms:** Provide Accession number in repository (where relevant) **OR** RRID		X

**Plants and microbes**	**Yes (indicate where provided: page no/section/legend)**	**n/a**
**Plants:** provide species and strain, unique accession number if available, and source (including location for collected wild specimens)		X
**Microbes:** provide species and strain, unique accession number if available, and source		X

**Human research participants**	**Yes (indicate where provided: page no/section/legend)**	**n/a**
Identify authority granting ethics approval (IRB or equivalent committee(s), provide reference number for approval.		X
Provide statement confirming informed consent obtained from study participants.		X
Report on age and sex for all study participants.		X

Design

**Study protocol**	**Yes (indicate where provided: page no/section/legend)**	**n/a**

For clinical trials, provide the trial registration number **OR** cite DOI in manuscript.		X

Laboratory protocol	Yes (indicate where provided: page no/section/legend)	n/a
Provide DOI or other citation details if detailed stepby-step protocols are available.	In the supplementary material file, the Materials and methods section includes the detailed description of each protocol used in the study. Further, references supporting the protocols were included.	

Experimental study design (statistics details)	Yes (indicate where provided: page no/section/legend)	n/a
State whether and how the following have been done**, or** if they were not carried out.		x
Sample size determination		X
Randomisation		X
Blinding		X
Inclusion/exclusion criteria		x

Sample definition and in-laboratory replication	Yes (indicate where provided: page no/section/legend)	n/a
State number of times the experiment was replicated in laboratory	The supplementary materials file includes the Materials and methods section described the replicates for each in vitro experiment and the numbers of samples used in each experiment.	
Define whether data describe technical or biological replicates	In vivo data represent biological replicates. Experiments in cell lines can be considered technical replicates, although efforts were made to perform everything independently (independent lentiviral transduction or DNA transfection). See Materials & Methods.	

Ethics	Yes (indicate where provided: page no/section/legend)	n/a
Studies involving human participants: State details of authority granting ethics approval (IRB or equivalent committee(s), provide reference number for approval.		X
Studies involving experimental animals: State details of authority granting ethics approval (IRB or equivalent committee(s), provide reference number for approval.	Materials and Methods: *"All in vivo experiments were approved by the Institutional Animal Care and Use Committee of the Broad Institute (Protocol #0162-05-16-2, most recent approval date: 2024-01-03) and were performed in accordance with the National Institutes of Health Guide for the Care and Use of Laboratory Animals."*	
Studies involving specimen and field samples: State if relevant permits obtained, provide details of authority approving study; if none were required, explain why.		X

Dual Use Research of Concern (DURC)	Yes (indicate where provided: page no/section/legend)	n/a
If study is subject to dual use research of concern, state the authority granting approval and reference number for the regulatory approval		X

Analysis

Attrition	Yes (indicate where provided: page no/section/legend)	n/a
State if sample or data point from the analysis is excluded, and whether the criteria for exclusion were determined and specified in advance.	No information was excluded during the analyzes.	X

Statistics	Yes (indicate where provided: page no/section/legend)	n/a
Describe statistical tests used and justify choice of tests.	Details pertaining to statistical tests are included in figure legends.	

Data Availability	Yes (indicate where provided: page no/section/legend)	n/a
State whether newly created datasets are available, including protocols for access or restriction on access.	Materials and methods: RNA-seq datasets are available on GEO (GSE255987)	
If data are publicly available, provide accession number in repository or DOI or URL.	Materials and methods: RNA-seq datasets are available on GEO (GSE255987)	
If publicly available data are reused, provide accession number in repository or DOI or URL, where possible.		X

Code Availability	Yes (indicate where provided: page no/section/legend)	n/a
For all newly generated code and software essential for replicating the main findings of the study:		
State whether the code or software is available.	https://github.com/edwin-n-neumann/CHARM_x_Prion	
If code is publicly available, provide accession number in repository, or DOI or URL.	https://github.com/edwin-n-neumann/CHARM_x_Prion	

Reporting

Adherence to community standards	Yes (indicate where provided: page no/section/legend)	n/a
MDAR framework recommends adoption of discipline-specific guidelines, established and endorsed through community initiatives. Journals have their own policy about requiring specific		

guidelines and recommendations to complement MDAR.		
State if relevant guidelines (eg., ICMJE, MIBBI, ARRIVE) have been followed, and whether a checklist (eg., CONSORT, PRISMA, ARRIVE) is provided with the manuscript.		X
